# Comparison of Luteal Support Protocols in Frozen IVF/ICSI Cycles: A Network Meta‐Analysis

**DOI:** 10.1111/1471-0528.18172

**Published:** 2025-05-02

**Authors:** Neerujah Balachandren, Meenakshi Veeramani, Sureka Suriyakumar, Sarah Wiley, Dimitrios Mavrelos, Ephia Yasmin, Stavroula L. Kastora

**Affiliations:** ^1^ University College London, UCL EGA Institute for Women's Health London UK; ^2^ School of Medicine Imperial College London London UK; ^3^ Department of Obstetrics and Gynaecology Barnet Hospital, Royal Free London NHS Foundation Trust London UK

**Keywords:** clinical pregnancy, fertility, frozen‐embryo transfer, live birth, luteal support, miscarriage, multiple pregnancies, network meta‐analysis

## Abstract

**Background:**

Luteal support is a core success factor of frozen embryo transfers (FET). However, inconsistency across recommended protocols generates notable heterogeneity across reproductive outcomes.

**Objective:**

To determine the most effective luteal support strategy (LPS) based on five key factors related to the effectiveness of FET cycles.

**Search Strategy:**

Twelve databases and two prospective registers were searched from inception to 1st January 2024. The study was prospectively registered under the PROSPERO database (CRD42024513549).

**Selection Criteria:**

Randomised Controlled Trials (RCTs) and observational studies of women undergoing frozen embryo transfers were included.

**Data Collection and Analysis:**

Bayesian network meta‐analysis (NMA) model presenting random effects, risk ratios (RRs) with 95% credibility intervals (CrIs) was employed. Primary outcomes included clinical pregnancy, live birth, and miscarriage. Secondary outcomes included biochemical pregnancy and multiple pregnancy events.

**Main Results:**

Fourteen studies, of which eight RCTs, comparing 12 interventions upon 4688 participants, were included. Overall, CiNeMa risk of bias was moderate, and network inconsistency per outcome was low. Thirteen studies reported on clinical pregnancy events with vaginal progesterone (VP) and a single or double dose of subcutaneous GnRH agonist (GnRHa), significantly improving clinical pregnancy, RR 1.86 [95% CrI 1.18, 2.93].

**Conclusions:**

The addition of 0.1 mg subcutaneous GnRH agonist in a single (Day 3 post ET) or double (Day 3 and Day 6 post ET) schema upon a vaginal progesterone regimen till Week 12 appears to improve clinical pregnancy events in FET cycles.

## 1 Introduction

The global application of frozen–thawed embryo transfer (FET) cycles has been prevalent since 1983, but over the past decade, the use of FETs has been exponentially rising, with FETs accounting for over a third of all IVF cycles in Europe and the United States. This trend can be primarily attributed to advancements in cryopreservation techniques, a rise in the adoption of single embryo transfer (SET) strategies, and the widespread use of preimplantation genetic testing. The efficacy of a FET cycle hinges on the synchronised development of the endometrium with those of the embryo to facilitate implantation. There are three main approaches for endometrial preparation: natural cycle FET (NC‐FET), artificial/programmed FET (AC‐FET) and cycles where follicular growth is induced through medication (stimulated/modified natural cycle FET).

The use of progesterone for luteal phase support (LPS) in ART has been widely investigated and we now have high‐quality evidence to support its use in both fresh and frozen cycles. However, there is an ongoing discussion regarding the optimal timing to initiate LPS and the most effective route, dose, and duration as well as the potential utility of supplementary agents including oestradiol, HCG (Human chorionic gonadotropin) and GnRHa (Gonadotropin‐Releasing Hormone agonist).

Till present, multiple meta‐analyses have attempted to explore the optimal LPS protocol for FET in view of favourable short and long‐term obstetric outcomes; however, given the plethora of suggested LPS protocols and the inherent variability of FET cycles, for example, natural, modified, or medicated, conclusive evidence remains scarce [1, 2, 3, 4]. Here we offer the first network meta‐analysis assessing the effectiveness of 12 LPS protocols, based on five key factors related to the effectiveness of ART: clinical pregnancy, live birth, miscarriage, biochemical pregnancy, and multiple pregnancy in frozen embryo transfer cycles.

## 2 Methods

**FIGURE 1:**
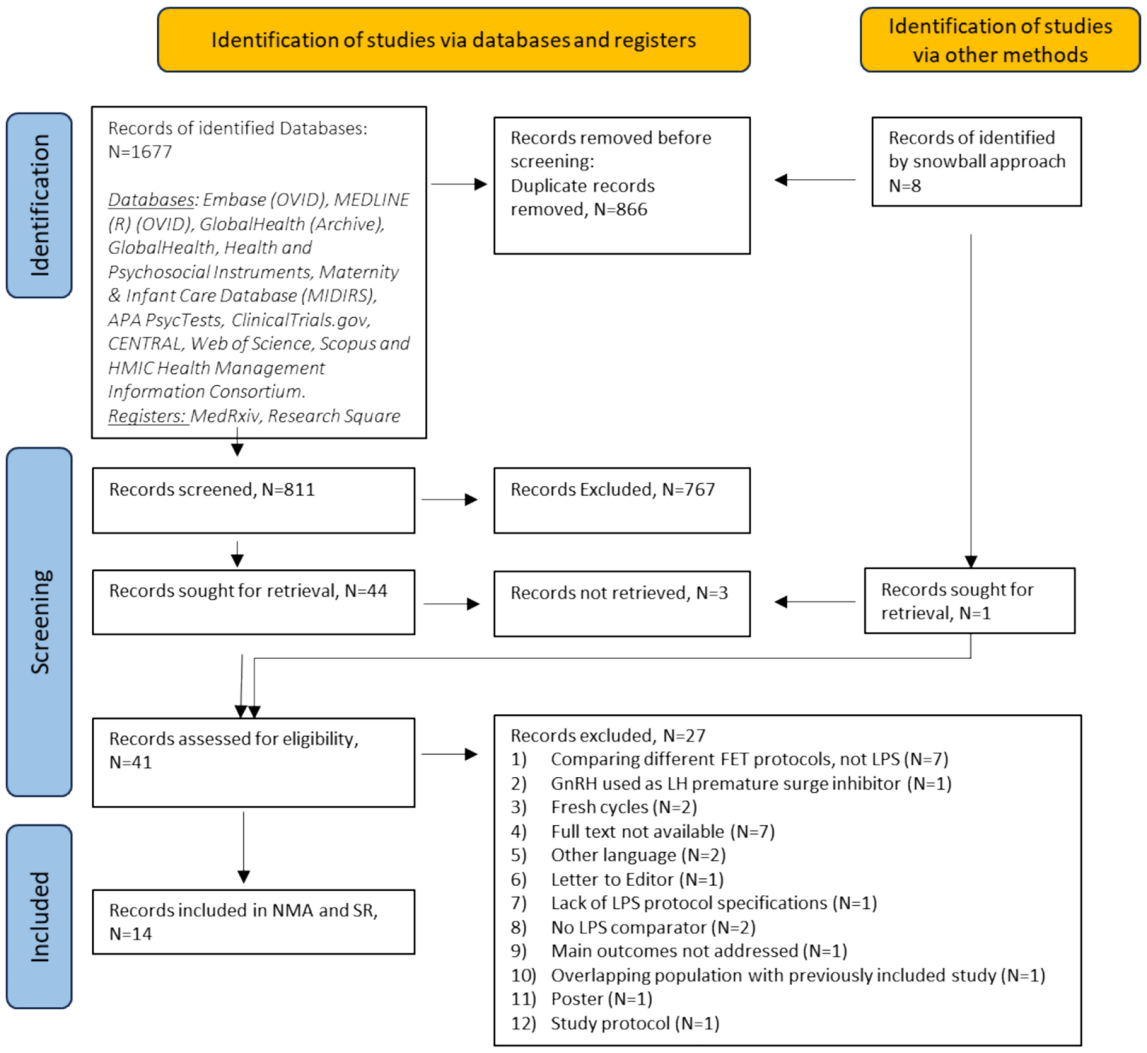
Prisma flow diagram.

Search strategy and selection criteria. The present study was prospectively registered under the PROSPERO database CRD42024513549 and conducted according to the PRISMA‐NMA checklist [5]. Twelve databases, namely Embase (OVID), MEDLINE (R) (OVID), GlobalHealth (Archive), GlobalHealth, Health and Psychosocial Instruments, Maternity & Infant Care Database (MIDIRS), APA PsycTests, ClinicalTrials.gov, CENTRAL, Web of Science, Scopus, and HMIC Health Management Information Consortium, and two prospective registers, MedRxiv and Research Square, were searched from inception to January 1st, 2024. The search strategy was as follows and adapted per the requirements of each target database (luteal and (support or supplementation or addition) and (assisted reproduction or IVF or ICSI or in vitro fertilisation) and frozen).mp. (mp = ti, ab, hw, tn, ot, dm, mf, dv, kf, fx, dq, cw, ta, te, bt, nm, ox, px, rx, an, ui, sy, ux, mx). To ensure that all previous meta‐synthesised evidence has been identified and assessed, a snowball approach has also been implemented, where the search of the databases described above was also conducted with a limit to include only meta‐analyses (*N* = 8). The original studies included in the relevant meta‐analysis manuscripts were extracted and deduplicated (*N* = 13). Those were compared to the manuscripts identified through the classical search (Figure 1). Observational and randomised control study designs were included in the initial search and downstream analyses. No language or geographical restrictions were applied.

For both systematic review and network meta‐analysis (NMA), studies comparing pharmacological treatments administered for luteal support, either as monotherapy or combinatorial therapy, against placebo or other active agents to women undergoing frozen IVF/ICSI cycles were included [6, 7]. Studies reporting outcomes from oocyte donation cycles, comparing dosage or timing of the same compound, or including patients that had undergone fresh embryo transfer, studies where the route or compound of LPS was not stated or ≥ 4 embryos transferred were excluded (Figure 1). Four independent researchers (NB, MV, SW, SLK) independently selected the studies, reviewed the main reports and Supporting Information, extracted the relevant information from the included trials, and assessed the risk of bias. Any discrepancies were double‐checked and resolved by discussion with other members of the review team (SS, DM). The list of excluded studies and justification can be found in Table S1.

### 2.1 Data Extraction

Events (%, *N*) of clinical pregnancy, live birth, biochemical pregnancy, miscarriage, multiple pregnancy and the total number of patients exposed per treatment were extracted. Patient demographics and treatment‐specific parameters were also collected to allow for NMA transitivity analysis and comprehensive exploration of employed treatments across studies. Crude data on patient age (Median, IQR), BMI (kg/m^2^) (Median, IQR), duration of infertility (years) (Median, IQR), percentage and crude number of individual patients (%, *N*) with a primary or secondary infertility diagnosis, basal AMH (ng/mL) (Median, IQR), basal LH (IU/L) (Median, IQR), basal FSH (IU/L) (Median, IQR), progesterone levels (ng/mL) (Median, IQR) on human gonadotrophin (hCG) trigger day or embryo transfer (ET) day, endometrial thickness (mm) (Median, IQR) on ET day and ovarian stimulation protocol (*N*; %), type of FET cycle (natural, modified and medicated) (*N*; %) were collected (Table 1; Figure S1–S3). Per study, the total percentage of frozen cycles, Day 3 ETs was calculated from the reported, individual study data. Missing SD or IQR were calculated from *p*‐values, *t*‐values, and standard error (SE) to allow for data harmonisation. When mean and standard deviation values were recorded, Bland's method was employed to calculate median and IQR [22]. Additionally, treatment‐specific parameters, namely active compound (Progesterone, Estradiol, hCG, GNRH‐a), brand name, route of administration (O, IM, SC, PV, Topical (Patch)) dose (Progesterone, Estradiol, and GnRH agonist in mg, hCG in IU), median day of treatment initiation and SD, median end of treatment (weeks) and SD, number of patients exposed to named compound (Figure S1–S3; Table 2) [23, 24]. Lastly, the number of oocytes retrieved and fertilisation rates (%) were extracted as reported per study, given the inclusion of ≥ 1 embryos per study and aggregate data analysed as descriptive statistics (Figure 3).

**TABLE 1:** Included studies. Patient sample, ovarian stimulation protocol, luteal support comparison and regimen.

Author et al., year	Country	Time period of study	Sample size (post‐drop outs)	Protocol for IVF (compound, dose, duration)	Treatments examined	Treatment 1	Treatment 2	Treatment 3
Eftekhar et al., 2013 [8]	Iran	2011 to 2012	102	**Agonist**: IMP (29, 56.9%), Placebo (31, 60.8%); **Antagonist**: IMP (22, 43.1%), Placebo (20, 39.2%)	IMP vs. Placebo	IMP; 100 mg, 36 h post trigger till 10 weeks, 51	Placebo; 51	N/A
Kim et al., 2014 [9]	Korea	2009 to 2012	228	**Agonist**: VP (34, 23.4%), Placebo (21, 25.3%); **Antagonist**: IMP (111, 76.6%), Placebo (62, 74.7%)	VP vs. Placebo	VP (gel) Crinone; 90 mg, 48 h post trigger till 12 weeks, 145	Placebo; 83	N/A
Schwartz et al., 2019 [10]	Germany	2016 to 2917	231	Not stated	VP vs. Placebo	VP (tablet); 400 mg, 48 h post trigger till 8 weeks, 123	Placebo; 108	N/A
Kyrou et al., 2010 [11]	Belgium	2006 to 2007	452	**Agonist**: VP (129, 53.1%), Placebo (107, 51.2%); **Antagonist**: VP (114, 46.9%), Placebo (53, 25.4%)	VP vs. Placebo	VP (tablet) (Utrogestan); 600 mg, 24 h post trigger till 7 weeks, 243	Placebo; 209	N/A
Lee et al., 2017 [12]	China	2013 to 2015	450	Not stated	IMHCG vs. Placebo	IMHCG (Brand not stated); 1500 IU, 2 doses, first on FET day and second 6 days post FET, 225	Placebo; 225	N/A
Lee et al., 2013 [13]	China	2009	408	Not stated	IMHCG vs. Placebo	IMHCG (Not stated); 1500 IU, 2 doses, first on FET day and second 6 dayspost FET, 225	Placebo; 225	N/A
Seikula et al., 2018 [14]	Finland	2013 to 2014	104	Not stated	SCGnRHa vs. Placebo	SCGnRHa (Triptorelin acetate Ferring); 1 dose, 0.1 mg 6 days post FET, 72	Placebo; 72	N/A
Ye et al., 2019 [15]	China	2015 to 2017	868	**Agonist**: SCGnRHa (358, 84.0%), Placebo (301, 83.4%); **Antagonist**: SCGnRHa (48, 11.3%), Placebo (45, 12.5%)	SCGnRHa vs. Placebo	SCGnRHa (Triptorelin acetate Ferring); 1 dose, 0.1 mg 3 days post FET, 426	Placebo; 361	N/A
Pabuccu et al., 2022 [16]	Turkey	2021 to 2022	163	Not stated	OP vs. VP vs. IMP	OP, Dydrogesterone (Duphaston), 40 mg, from when endometrial thickness ≥ 8 mm up till 12 weeks, 52	VP (gel), Progesterone Vaginal Gel (Crinone 8%, Serono, Switzerland), 180 mg, from when endometrial thickness ≥ 7 mm up till 12 weeks, 55	IMP, Progestan (oil), 100 mg, from when endometrial thickness ≥ 7 mm up till 12 weeks, 44
Vuong et al., 2021 [17]	Vietnam	2019 to 2020	1364	Not stated	VP vs. OP+VP	VP, (pessary) (Cyclogest, Actavis, UK) 1600 mg, from when endometrial thickness ≥ 8 mm up till 7 weeks, 632	OP + VP (pessary), Dydrogesterone, (Duphaston; Abbott), 20 mg; Micronized Vaginal Progesterone (Cyclogest; Actavis), 800 mg, from when endometrial thickness ≥ 8 mm up till 7 weeks, 732	N/A
Zareii et al., 2021 [18]	Iran	2016 to 2017	240	Both agonist & antagonist protocols—numbers of each not stated	VP vs. VP + SCGnRHa	VP (pessary) (Cyclogest, Actavis, UK), 1600 mg () started after ET (day not defined),120	VP (pessary) + SCGnRHa, vaginal progesterone (Cyclogest, Actavis, UK) 800‐mg, started after ET & SC GnRH‐agonist (triptorelin or dipherelin; Decapeptyl; IPSN, France), 0.1 mg started on day of ET, and then 3 and 6 days after ET, 120	N/A
Ozer et al., 2021 [19]	Turkey	2018 to 2019	134	Not stated	VP vs. OP	VP (gel), (Crinone 8%, Serono, Switzerland), 90 mg, 36 h after r‐hCG trigger and continued until the 12 weeks, 67	OP, oral dydrogesterone (Duphaston, Abbott Healthcare Products), 30 mg, started 36 h after r‐hCG trigger and continued up till 12 weeks, 67	N/A
Shiotani et al., 2017 [20]	Japan	2003 to 2004	173	Not stated	TE + VP + IMHCG vs. TE + VP	TE + VP + IMHCG, Transdermal Estradiol (Estraderm M; Kissei Pharm, Tokyo, Japan) step‐up regime (2.16–4.32 mg) started on day 2 of cycle and continued after ET. Vaginal progesterone suppository, 600 mg, commenced on day 15 and continued after ET. ET on day 17 or day 20 of cycle. IM HCG 3000 IU administered on day 17, 20 and 23, 86.	N/A	N/A
Davar et al., 2015 [21]	Iran	2014 to 2015	200	Not stated	OE + VP vs. OE + VP + SCGnRHa	OE + VP (pessary), Estradiol valerate 6 mg from Day 2 of cycle and continued after ET. Once endometrial thickness ≥ 8 mm addition of VP (suppository) 800 mg and continued after ET (duration not defined), 100	N/A	N/A

**TABLE 2:** LPS protocol characteristics per compound. Compound mono‐ or multi‐treatment for luteal support, median and maximum dose, median day of luteal support initiation, and median duration of treatment. Dosage in milligrams (mg) unless otherwise stated.

Route of administration	Compound	Brand Name	Dose (mode and range) mg	Median start of LPS (SD)	Median end of treatment (SD)	Number of patients
Vaginal	Progesterone (Gel)	Crinone	90 (90, 180)	ETs ≥ 7 mm	12 (10,12)	267
Vaginal	Progesterone (Tablet)	Utrogestan; Cyclogest	800 (400, 800)	At embryo transfer OR once ET ≥ 8 mm	7 (7, 8)	1525
Oral	Progesterone (Tablet)	Duphaston	40 (20, 40)	Once ET ≥ 7 mm OR ≥ 8 mm	12 (7,12)	851
Intramuscular	Progesterone (oil)	Progestan	100	Once ET ≥ 7 mm	10 (10,12)	95
Transdermal	Estradiol (Patch)	Estraderm M	Step‐up regime (2.16–4.32)	D2 of cycle and continued after embryo transfer	Not stated	173
Oral	Estradiol (Oral)	Not stated	6	D2 of cycle and continued after embryo transfer	Not stated	200
Subcutaneous	GnRH agonist (solution)	Triptorelin acetate; Dipherelin; Decapeptyl	0.1	D3 OR D6 post embryo transfer (single dose) OR D3 + D6 (two doses)	1 OR 2 doses	818
Intramuscular	Human Chorionic Gonadotrophin (solution)	Not stated	1500 IU (1500, 3000)	D0 and D6 OR D3 post embryo transfer	2 doses	514

### 2.2 Outcomes

The NMA primary outcomes were clinical pregnancy, defined as the presence of a gestational sac, with or without a fetal heartbeat on ultrasonography (US) at 6 weeks of gestation and confirmed by human chorionic gonadotropin (hCG) assay [25], live birth, defined as the number of deliveries that resulted in live born neonate/s, and miscarriage defined as the spontaneous loss of a pregnancy before the 20th week. Regarding live birth, singleton and non‐singleton deliveries were considered as a single event. Secondary outcomes included biochemical pregnancy, defined as a positive hCG test but without US verification 2 weeks following embryo transfer (ET), and multiple pregnancy was defined as non‐singleton clinical pregnancy. Crude events were collected per included study, and therefore no homogenisation of extracted data was required.

### 2.3 Data Analysis

Effect estimates were calculated as risk ratios (RRs) for all outcomes, given that all were dichotomous, with respective 95% credibility intervals (95% CrIs) using Bayesian network meta‐analysis [26] (Figure 5, Figures S4–S6). Of note, a credibility interval is an interval within which an unobserved parameter value falls with a particular probability in Bayesian statistics comparable to the 95% Confidence interval commonly seen in frequentist statistics [27]. Network meta‐analysis iterations were conducted with the MetaInsight visual R package [28]. NMA was conducted using a random‐effects model within a Bayesian setting, as unequal heterogeneity across all comparisons was assumed. Placebo was used as the reference treatment. A hierarchy of treatments was calculated for each outcome, based on the p‐scores and SUCRA ratings. Summary of the rank distribution of LPS treatments, interpreted as the estimated proportion of treatments worse than placebo, was displayed by Litmus Rank‐O‐Gram graphs and Radial SUCRA [29] (Figures S7 and S8). Transitivity assumption was evaluated by comparing the distribution of key study characteristics across studies grouped by comparison (type of FET protocol and design of study) (Figures S9 and S10). We assessed inconsistency between direct and indirect sources of evidence using global and local approaches. We assessed global inconsistency by using a design‐by‐treatment test [30, 31]. Local inconsistency was evaluated by using the back calculation and separate indirect from direct design evidence methods, comparing direct and indirect evidence for each pairwise treatment comparison and node‐splitting model [32] (Table S7). Possible heterogeneity of treatment effects and the robustness of findings were explored by subgroup network meta‐analyses including only trials at overall low and medium risk of bias. Further subgroup analysis was conducted on only RCT design studies and trials of natural FET cycle. Subgroup analyses of other types of cycles, namely medicated or natural‐modified, were not feasible due to the limited number of studies exploring these protocols, limiting the option of NMA. Gelman network convergence, network deviance, and ranking analysis were conducted to quantify overall network discordance (Figures S9 and S10). Intergroup differences regarding demographic and treatment parameters were quantified, where appropriate, by ANOVA (for parametric distributed variables e.g., Age, BMI) or Kruskal‐Wallis test (non‐parametric distribution of variables, e.g., all remaining variables). Only statistically significant differences were displayed in graphs in a numerical format. Descriptive statistics and relevant bar charts were generated by the GraphPad Prism V. 10 academic licence [33].

### 2.4 Risk of Bias Assessment

Within‐study bias was assessed with the Cochrane risk of bias tool RoB2 [34] and Robins‐I tool [35] for observational studies (Tables 3 and 4). Overall network risk of bias was assessed with the Network Meta‐Analysis framework (CINeMA) [36] (Tables S5–S7). Small‐study effects and publication bias for each treatment pair were assessed using a contour‐enhanced funnel plot. Certainty in evidence was assessed by the GRADE framework (Table 5) [37].

**TABLE 3:** Revised Cochrane risk‐of‐bias tool for randomised trials (RoB 2) assessment per RCT studies.

	Revised Cochrane risk‐of‐bias tool for randomised trials (RoB 2)						
Study	Domain 1: Risk of bias arising from the randomization process	Domain 2: Risk of bias due to deviations from the intended interventions	Domain 2: Risk of bias due to deviations from the intended interventions (effect of adhering to intervention)	Domain 3: Missing outcome data	Domain 4: Risk of bias in measurement of the outcome	Domain 5: Risk of bias in selection of the reported result	Overall risk of bias
Eftekhar et al., 2013 [8]	Low	Some Concerns	Low	Low	Low	Low	Low
Lee et al., 2017 [12]	Low	Low	Low	Low	Low	Low	Low
Ye et al., 2019 [15]	Low	Low	Low	Low	Low	Low	Low
Pabuccu et al., 2022 [16]	Low	Low	Some concerns	Low	Low	Some concerns	Low
Zareii et al., 2021 [18]	Low	Low	Low	Some concerns	Some concerns	Some concerns	Low
Ozer et al., 2021 [19]	Some concerns	Some concerns	Low	Some concerns	Low	Low	Low
Shiotani et al., 2017 [20]	Some concerns	Major concerns	Some concerns	Major concerns	Some concerns	Some concerns	Low
Davar et al., 2015 [21]	Some concerns	Major concerns	Some concerns	Some concerns	Some concerns	Some concerns	Low

**TABLE 4:** ROBINS‐I tool risk of bias assessment per observational studies.

Study	ROBINS‐I tool						
	Pre‐intervention domains/bias due to confounding	Pre‐intervention domains/bias in selection of participants into the study	At‐intervention domain/bias in classification of interventions	Post‐intervention domains/bias due to deviations from intended interventions	Post‐intervention domains/bias due to missing data	Post‐intervention domains/bias in measurement of the outcome	Post‐intervention domains/bias in selection of the reported result
Kim et al., 2014 [9]	Low	Low	Low	Low	Moderate	Low	Low
Schwartz et al., 2019 [10]	Moderate	Moderate	Low	Low	Moderate	Low	Low
Kyrou et al., 2010 [11]	Low	Moderate	Low	Low	Low	Low	Moderate
Lee et al., 2013 [13]	Moderate	Moderate	Low	Moderate	Moderate	Low	Moderate
Seikula et al., 2018 [14]	Moderate	Low	Moderate	Low	Low	Moderate	Moderate
Vuong et al., 2021 [17]	Low	Low	Low	Low	Low	Low	Low

**TABLE 5:** GRADE certainty in evidence ratings per study.

Study		GRADE					
		Risk of bias	Imprecision	Inconsistency	Indirectness	Publication bias	Collectively
Eftekhar et al., 2013 [8]	RCT	⊕⊕⊕⊕	⊕⊕⊕◯	⊕⊕⊕⊕	⊕⊕⊕⊕	⊕⊕⊕⊕	⊕⊕⊕⊕
Kim et al., 2014 [9]	Retrospective	⊕⊕⊕◯	⊕⊕⊕◯	⊕⊕⊕◯	⊕⊕⊕◯	⊕⊕⊕◯	⊕⊕⊕◯
Schwartz et al., 2019 [10]	Retrospective	⊕⊕⊕◯	⊕⊕⊕◯	⊕⊕⊕⊕	⊕⊕⊕◯	⊕⊕⊕◯	⊕⊕⊕◯
Kyrou et al., 2010 [11]	Retrospective	⊕⊕⊕⊕	⊕⊕⊕◯	⊕⊕⊕⊕	⊕⊕⊕◯	⊕⊕⊕⊕	⊕⊕⊕⊕
Lee et al., 2017 [12]	RCT	⊕⊕⊕⊕	⊕⊕⊕⊕	⊕⊕⊕⊕	⊕⊕⊕⊕	⊕⊕⊕⊕	⊕⊕⊕⊕
Lee et al., 2013 [13]	Retrospective	⊕⊕⊕◯	⊕⊕⊕◯	⊕⊕◯◯	⊕⊕⊕◯	⊕⊕⊕◯	⊕⊕⊕◯
Seikula et al., 2018 [14]	Prospective	⊕⊕⊕◯	⊕⊕⊕◯	⊕⊕⊕⊕	⊕⊕⊕◯	⊕⊕⊕⊕	⊕⊕⊕⊕
Ye et al., 2019 [15]	RCT	⊕⊕⊕⊕	⊕⊕⊕⊕	⊕⊕⊕⊕	⊕⊕⊕⊕	⊕⊕⊕⊕	⊕⊕⊕⊕
Pabuccu et al., 2022 [16]	RCT	⊕⊕⊕⊕	⊕⊕⊕◯	⊕⊕⊕◯	⊕⊕⊕⊕	⊕⊕⊕⊕	⊕⊕⊕⊕
Vuong et al., 2021 [17]	Prospective	⊕⊕⊕◯	⊕⊕⊕◯	⊕⊕⊕◯	⊕⊕⊕⊕	⊕⊕⊕⊕	⊕⊕⊕◯
Zareii et al., 2021 [18]	RCT	⊕⊕⊕◯	⊕⊕⊕◯	⊕⊕⊕◯	⊕⊕⊕◯	⊕⊕⊕⊕	⊕⊕⊕◯
Ozer et al., 2021 [19]	RCT	⊕⊕◯◯	⊕⊕⊕◯	⊕⊕⊕◯	⊕⊕⊕◯	⊕⊕⊕◯	⊕⊕⊕◯
Shiotani et al., 2017 [20]	RCT	⊕⊕⊕◯	⊕⊕⊕◯	⊕⊕◯◯	⊕⊕◯◯	⊕⊕⊕◯	⊕⊕◯◯
Davar et al., 2015 [21]	RCT	⊕⊕⊕◯	⊕⊕⊕◯	⊕⊕⊕◯	⊕⊕⊕◯	⊕⊕⊕⊕	⊕⊕⊕◯

## 3 Results

### 3.1 Included Study Design and Quality of Evidence Assessment

From 1685 records initially retrieved from the search results, 14 studies, of which 8 RCTs, comparing 12 interventions, and including 4688 participants, were integrated in the present NMA (Figure 1; Table 1). RCT study risk of bias was assessed by the RoB‐2 tool and was deemed low (Table 3) while observational study risk of bias was assessed by the Robins‐I tool and was deemed moderate (Table 4). NMA risk of bias was assessed through the CiNeMa tool per outcome and was deemed moderate (Tables S2–S6), while network inconsistency per outcome was deemed low (Clinical Pregnancy χ^2^: 0.48, Live Birth χ^2^: 0.53, Miscarriage χ^2^: 0.81, Biochemical Pregnancy χ^2^: 0.26) and moderate (Multiple Pregnancy χ^2^: 2.14). Overall, GRADE confidence in evidence was deemed “high” for six studies, “moderate” for seven studies, and “low” for one study (Table 5).

### 3.2 Participant and Treatment Characteristics

A total of 4688 participants were assigned to any of the following 12 interventions: placebo (no exposure) (*N* = 1334), VP (vaginal progesterone) (*N* = 820), IMP (intramuscular progesterone) (*N* = 95), OP (oral progesterone) (*N* = 186), IMHCG (intramuscular hCG) (*N* = 430), SCGNRH‐a (subcutaneous GNRH agonist) (*N* = 498), OP+VP (oral progesterone and vaginal progesterone) (*N* = 732), VP + SCGNRH‐a (vaginal progesterone and subcutaneous GNRH agonist) (*N* = 220), OE + VP (oral estradiol and vaginal progesterone) (*N* = 100), TE + VP (Transdermal Oestrogen and vaginal progesterone) (*N* = 87), OE + VP + SCGnRHa (Oral estradiol, vaginal progesterone and subcutaneous GNRH agonist) (*N* = 100) and TE + VP + IMHCG (Transdermal Oestrogen, vaginal progesterone and intramuscular HCG) (*N* = 86) (Tables 1 and 2). The most employed dose of vaginal progesterone was 800 mg (range 400, 800) in a tablet formulation, while 90 mg (90, 180) in a gel formulation (Table 2). Regarding subcutaneous GNRH agonist administration, the dosage regimen included a once‐only or twice‐only administration of 0.1 mg Triptorelin acetate, Dipherelin, or Decapeptyl at Day 3 post FET, and if a 2‐dose protocol was followed, a subsequent dose on Day 6 post FET (Table 2). LPS in the form of vaginal progesterone was initiated once endometrial thickness was ≥ 7 mm (7, 8 mm) or at embryo transfer and continued till 12 weeks (10, 12) if in the form of gel, or 7 weeks (7, 8) if in the form of tablet.

**FIGURE 2:**
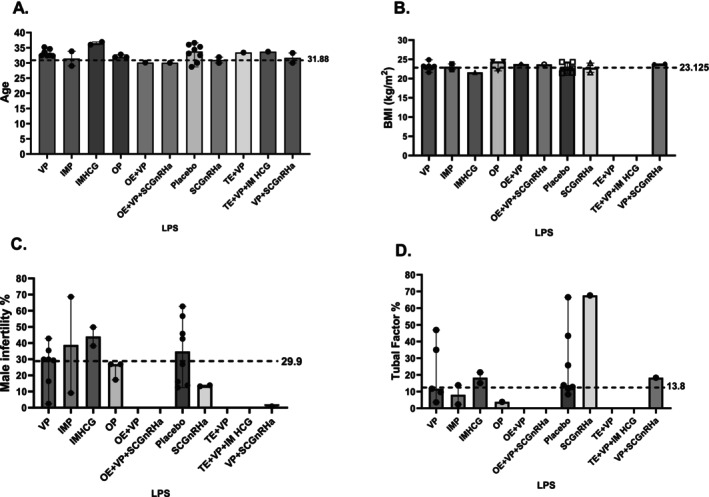
Population percentage and crude numbers exposed to each luteal support regimen and baseline demographic characteristics.

**FIGURE 3:**
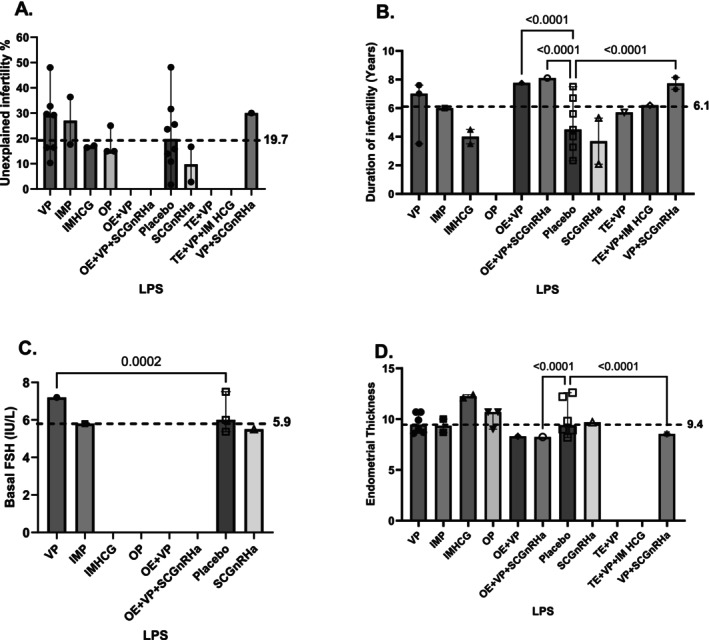
Population percentage and crude numbers exposed to each luteal support regimen and baseline clinical characteristics.

The placebo exposed group was considered as the control cohort given that most included studies employed this as the control (Table 1). Median participant age across all treatment groups was 31.88 years [IQR 31.55, 33.4] (Figure 2A) and the median BMI was 23.12 (kg/m^2^) [IQR 21.05, 24.9] (Figure 2B). Inter‐treatment group age and BMI differences were statistically quantified and not found to be statistically significant against the placebo cohort. Median male factor infertility percentage across reported interventions was at 29.9% (range 2.2%, 69.7%) (Figure 2C) while tubal factor infertility median was at 13.8% (range 1.8%, 70%) (Figure 2D). The percentage of unexplained infertility as an indication for IVF/ICSI was reported at 19.7% (range 3.2%, 49.6%) across comparator groups (Figure 3A) while the median duration of infertility prior to IVF/ICSI intervention was 6.1 years [IQR 3.35, 7.4] (Figure 3B). Duration of infertility was found to be significantly different in the OE + VP (median 7.76 years), OE + VP + SCGnRHa (median 8.1 years) and VP + SCGnRHa (median 7.72 years) groups. Median basal FSH was found to be 5.9 (IU/mL) [IQR 5.5, 7.5] however consistently under‐reported across studies (Figure 3C) while the endometrial thickness median (mm) on the day of embryo transfer was 9.4 mm [IQR 8.2, 13.2] and significantly different in comparison to the control group between OE + VP + SCGnRHa (median 8.24 mm) and VP + SCGnRH (8.54 mm) (Figure 3D).

In regard to day of embryo transfer, a median of 51.48% (Mean: 45.58%; Range 0, 100) ETs were of Day 5 embryos; however, this variable was reported by approximately half of the included studies [8, 9, 10, 11, 13, 14, 16, 17] (Figure S1A). Additionally, transferred embryo quality was reported by 9 studies [8, 9, 12, 15, 16, 17, 18, 19, 21] with a median of 58.4% embryos transferred (Mean: 62.82; Range 47%, 100%) being reported as I or II quality (good) (Figure S1B). A 34.45% of the included cohort underwent a natural FET, 59.47% medicated, and 6.21% modified natural cycle (Figure S1C). The median follow‐up following embryo transfer was at 20 months (range 20, 40) (Figure S1D), Median fertilisation rate was at 69.58%, while the median number of oocytes retrieved was 13 [IQR 5, 16] (Figure S3A,B).

### 3.3 Data Synthesis and Network Meta‐Analysis

**FIGURE 4:**
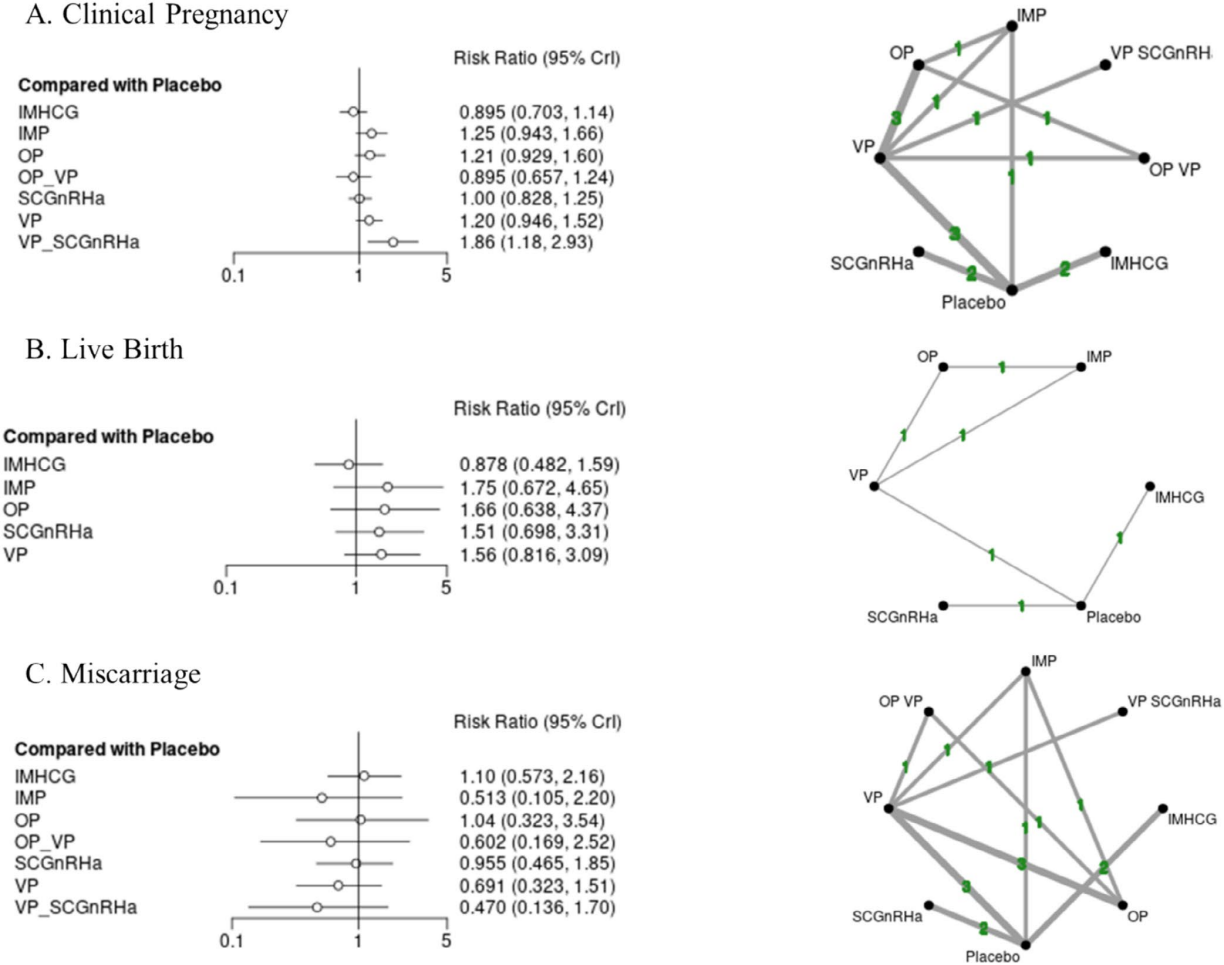
Luteal support Bayesian random effect consistency forest plot (risk ratio, 95% CrI) for clinical pregnancy (A) live birth (B) miscarriage (C) outcomes.

**FIGURE 5:**
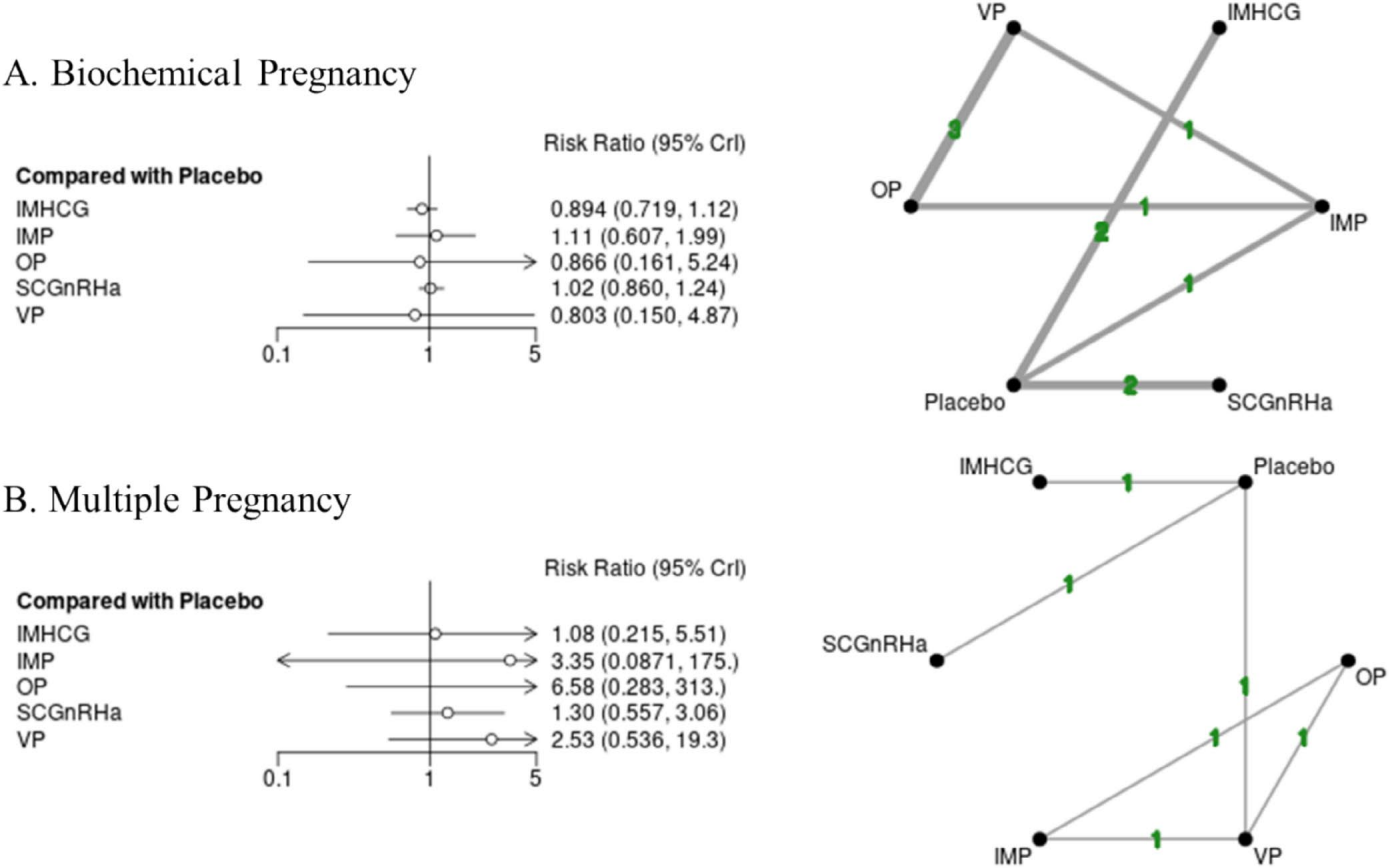
Luteal support Bayesian random effect consistency forest plot (risk ratio, 95% CrI) for biochemical pregnancy (A) and multiple pregnancy (B) outcomes.

Placebo was considered as the reference treatment across all outcomes (Figures 4 and 5; Figures S4–S6; Tables S2–S7). Regarding NMA primary outcomes, clinical pregnancy events were reported by 13 studies, and CiNeMa NMA RoB rating was deemed “moderate” (Table S2). For Placebo vs. all other LPS protocols, overall network incoherence was found to be low, *χ* ^2^ 0.485, 3° of freedom, *p*‐value: 0.92 (Figure 4A; Figure S8A; Table S7). VP + SCGnRHa appeared to be the only LPS regimen with resulting in improved clinical pregnancy events [RR 1.86 (95% CrI 1.18–2.93)] (Figure 4; Figure S7). Subgroup analysis of natural cycles alone was not feasible for VP + SCGnRHa given network disconnection; however, RCT‐only subgrouping highlighted a similarly significant benefit in regard to clinical pregnancy events [RR 2.25 (95% CrI 1.13–4.62)] (Figure S4A). Subgroup analysis of medicated cycles again highlighted VP + SCGnRHa as a superior LPS in view of improved clinical pregnancy events [RR 2.52 (95% CrI 1.35–4.89)] (Figure S6A).

In regard to live pregnancy events, as reported by 6 studies, CiNeMa NMA RoB rating was deemed “moderate” (Table S3) and overall network incoherence was found to be low, *χ* ^2^ 0.53 (5° of freedom), *p*‐value: 0.82 (Figure 4B; Figure S8B; Table S3). None of the interventions were found to be significantly superior to placebo in either aggregate or subgroup analyses (Figure 4B; Figures S5B, S6B, S7). In regard to miscarriage events, reported by 14 studies, CiNeMa NMA RoB rating was deemed “high” (Table S4) and overall network incoherence was found to be low *χ* ^2^ 0.81 (3° of freedom), *p*‐value: 0.74 (Figure 4C; Figure S8C; Table S4). None of the LPS assessed in the NMA, were found to be significantly reducing miscarriage events in either aggregate or subgroup analyses (Figure 4C; Figures S4C and S6C). However, miscarriage events were reduced in the VP + SCGnRHa cohort, a result that did not reach statistical significance [RR 0.47 (95% CrI 0.14 to 1.70)]. In regard to secondary outcomes, none of the LPS regimens assessed appeared to increase biochemical pregnancy or multiple pregnancy events in aggregate or subgroup analyses (Figure 5A,B; Figures S5, S8–S10; Tables S5 and S6).

Overall, the present NMA highlights vaginal progesterone with the addition of subcutaneous GnRH agonist as a single (Day 3 post‐FET) or double (and on Day 6 post FET) dose, which may improve clinical pregnancy outcomes, especially in medicated frozen cycles whilst reducing miscarriage events, albeit the latter not reaching statistical significance.

## 4 Discussion

### 4.1 Main Findings

The present NMA of 4688 participants, examining 12 LPS interventions including placebo, suggests that the addition of 0.1 mg subcutaneous GnRH agonist in a single (Day 3 post embryo transfer) or double (Day 3 and Day 6 post embryo transfer) schema upon a vaginal progesterone regimen (Crinone, 90 mg (range 90, 180) or Utrogestan, 800 mg (range 400, 800)), appears to significantly improve clinical pregnancy events in frozen embryo transfer, and more specifically in medicated cycles.

### 4.2 Interpretation

To facilitate the process of embryo transfer, endometrial preparation is necessary to enable implantation in medicated cycles. In turn, in a frozen cycle, it is necessary to optimise endometrial preparation with the administration of oestrogens, followed by the introduction of progestogens with a plethora of available protocols [38]. Moreover, the initiation of luteal phase support following or prior to embryo transfer can be initiated and subsequently stopped at varied time intervals, with similar variability in the respective LPS included compounds [39]. While the optimisation of frozen embryo transfer cycles holds significant importance given to the percentage increase of FET cycles by over 18% from 2019 to 2021, the paucity of data in view of the optimal LPS to support such transfers is notable [6, 40, 41, 42]. Previous meta‐analyses have suggested that luteal GnRH‐a treatment may improve clinical pregnancy events [40, 42, 43]. However, inherent to the limitations of a pairwise meta‐analysis, the control group was substantially divergent, incorporating patients that had received vaginal progesterone and occasionally a single dose of hCG [44, 45, 46]. In this context, the performance of other LPS protocols would be challenging to discern.

GnRH agonist either as a single bolus or as a dual bolus has been shown to exert a direct effect upon both the embryo and the placenta to induce hCG synthesis; however, in the context of FET as an LPS regimen, its mechanism of function remains obscure [42, 47, 48]. One may hypothesise that GnRH agonist administration may facilitate interaction between the endometrium and trophoblast, supporting embryo implantation through proangiogenic factor release [14, 49]. GnRH agonists may also restore LH levels and support the luteal phase naturally. Nonetheless, the mechanism of action and possibly synergistic effects with concurrent vaginal progesterone administration need to be further evaluated. Importantly, previous meta‐synthesised evidence examining the effect of GnRH agonist as an LPS in fresh cycles did not identify any association between the said LPS and fetal malformations [50].

However, LPS cannot be administered in a “one size fits all” approach and important aspects to be considered include the type of FET protocol followed, namely natural, modified or medicated as well as LPS administration caveats that may impact upon LPS effectiveness. In the present study, we explored the optimal LPS in natural and medicated FET cycles. While cumulative evidence suggested the superiority of VP + SCGnRHa regarding clinical pregnancy outcomes [51], the finding remained statistically significant only in medicated cycles. Importantly, no inferences could be drawn regarding VP + SCGnRHa in natural cycles due to network disruption suggesting that further studies would be necessary reach definitive conclusions in respect to its effects upon clinical pregnancy in natural FET. Notably, incorporation of GnRHa as an LPS protocol would result in a single or double subcutaneous administration of an additional agent upon a standardised vaginal progesterone (Crinone, mode 90 mg (range 90, 180) or Utrogestan, mode 800 mg (range 400, 800)) LPS. As such, the acceptability of this approach, should be primarily discussed with the patient, empowering shared decision making and compliance with treatment [52].

Another parameter for consideration remains the variability across initiation, cessation, and dosage of LPS, which is notable across both fresh and frozen cycles [49]. Variations in timing, dosage, and duration of progesterone administration can lead to suboptimal endometrial receptivity, resulting in implantation failure or miscarriage [53]. Inconsistent practices complicate comparative studies, hindering evidence‐based recommendations [54]. Moreover, excessive or prolonged progesterone use increases patient burden, costs, and side effects, reducing compliance and overall satisfaction [55]. Uniform guidelines are essential to optimise outcomes, improve consistency, and enhance patient‐centered care in FET cycles. In this work, we aimed to summarise available evidence and to provide the most commonly employed dosage and duration LPS regimens (Table 2). As such, the evidence provided herein can be translated to clinical practice as both comparators and leads by which individual reproductive medicine units can both review their respective LPS dosages against published literature and decide upon possibly a lower, more cost‐effective but equally efficient dosage. The latter, in the context of medication rationalisation, remains a modern medicine challenge and call for action [56].

### 4.3 Strengths and Limitations

The investigation of the most effective protocol for luteal support is an ongoing and dynamic area of study within the realm of artificial reproduction. Given the wide range of LPS protocols that are now available, the precision of estimations provided by NMA offers a more thorough perspective on the relative effectiveness of various interventions. The current study is the first network meta‐analysis in the field of LPS in FET, providing insights not only upon the optimal LPS across five main outcomes but also the expected administration dosage and duration of such regimen. Additionally, sensitivity analysis was conducted to further investigate confounding factors and identify sources of heterogeneity by focusing on specific types of frozen cycles (natural or medicated) and the design of the included studies. Importantly, the number of available studies reporting on natural or modified natural cycles limited the feasibility of subgroup analyses in the context of clinical pregnancy outcomes, and in turn, results should be taken with caution.

In the present study, LPS protocols have been considered as distinct entities for comparison, enabling the evaluation of specific outcomes at the protocol level rather than the compound level. Nevertheless, the evaluation of side effects and safety characteristics of combinatorial treatments has not been conducted, therefore posing a substantial factor that influences tolerance and compliance, particularly in the context of luteal support protocols. Consequently, further investigation is required to address this issue. Furthermore, the absence of a cost‐effectiveness analysis necessitates its inclusion in a comprehensive decision‐making process that involves both patients and clinicians. Lastly, it should be noted that while the current study reports on dosage, duration, and timing of LPS, this was not its primary objective. Therefore, in order to provide specific recommendations about these parameters of LPS, it is recommended that further studies with relevant designs be conducted.

## 5 Conclusion

The present study presents the first NMA in the field of LPS usage in FET. Findings suggest that the combination of 0.1 mg subcutaneous GnRH agonist in a single (Day 3 post embryo transfer) or double (Day 3 and Day 6 post embryo transfer) schema with vaginal progesterone continuing for up to 12 weeks appears to significantly improve clinical pregnancy events in frozen embryo transfer cycles whilst associating with a reduction in miscarriage events. Notably, the observed reduction in miscarriage events did not reach statistical significance and, as such, further studies are required to provide robust grounds for clinical recommendations.

## Author Contributions

Study concepts: S.L.K., N.B. Study design: S.L.K. Data acquisition: N.B., M.V., S.W., S.L.K. Quality control of data and algorithms: S.S., S.L.K. Data analysis and interpretation: N.B., S.S., D.M., S.L.K. Statistical analysis: S.L.K. Manuscript preparation: S.L.K. Manuscript editing: N.B., M.V., S.S. Manuscript review: D.M., S.L.K.

## Disclosure

Data regarding any of the subjects in the study have been published in the form of randomised controlled studies. Crude data were extracted and homogenised for the purposes of the present systematic review and network meta‐analysis. All included studies have been referenced as required.

## Conflicts of Interest

The authors declare no conflicts of interest.

## Supporting information


Tables S1–S7.

Figures S1–S10.

## Data Availability

All data associated with the present study are available in the main body or the Supporting Information of the submission.
