# Supplementary material for: Comparison of Luteal Support Protocols in Frozen IVF/ICSI Cycles: A Network Meta‐Analysis
Source: BJOG. 2025 May 2;132(9):1187–201. doi: 10.1111/1471-0528.18172 (PMC12232591; doi:10.1111/1471-0528.18172)
Supplement: Supplementary file 1 — Tables S1–S7. [file BJO-132-1187-s001.docx]

| **Author; Year** | **Reason of exclusion** |
| --- | --- |
| Queenan et al., 1994 | Comparing different FET protocols |
| Tanos et al., 1996 | Comparing different FET protocols |
| Dal Prato et al., 2002 | Comparing different FET protocols |
| El Toukhy et al., 2004 | Comparing different FET protocols |
| Simon et al., 1998 | Comparing different FET protocols |
| Tomax et al., 2012 | Comparing different FET protocols |
| Davar et al., 2015 | Comparing different FET protocols |
| Isik et al., 2009 | Examining GnRHa as prevention of a premature LH surge, not as LPS |
| Haahr et al., 2017 | Fresh cycles |
| Tesarik et al., 2006 | Fresh cycles |
| Sun et al., 2017 | Full text not available |
| Check et al., 2010 | Full text not available |
| Lan et al., 2007 | Full text not available |
| Sarfai et al., 2023 | Full text not available |
| Torelli et al., 2016 | Full text not available |
| Svenstrup et al., 2022 | Full text not available |
| Ben-Ami et al., 2015 | Full text not available |
| Deng et al., 2021 | Language: Chinese |
| Gogce et al., 2015 | Language: French |
| Ata and Urman, 2010 | Letter to Editor |
| Wang et al., 2021 | No details regarding the LPS protocol (dosage and route of administration) |
| Aydin et al., 2022 | No LPS comparator |
| Hershko Klement et al., 2016 | Outcomes not addressed |
| Li et al., 2017 | Population overlaps with Lee et al., 2017 (included) |
| Hokenstad et al., 2013 | Poster |
| Nayar et al., 2022 | No LPS comparator |
| Jiang et al., 2022 | Study protocol published; study not complete |

**Table S1.** Excluded studies (N=27) and justification.


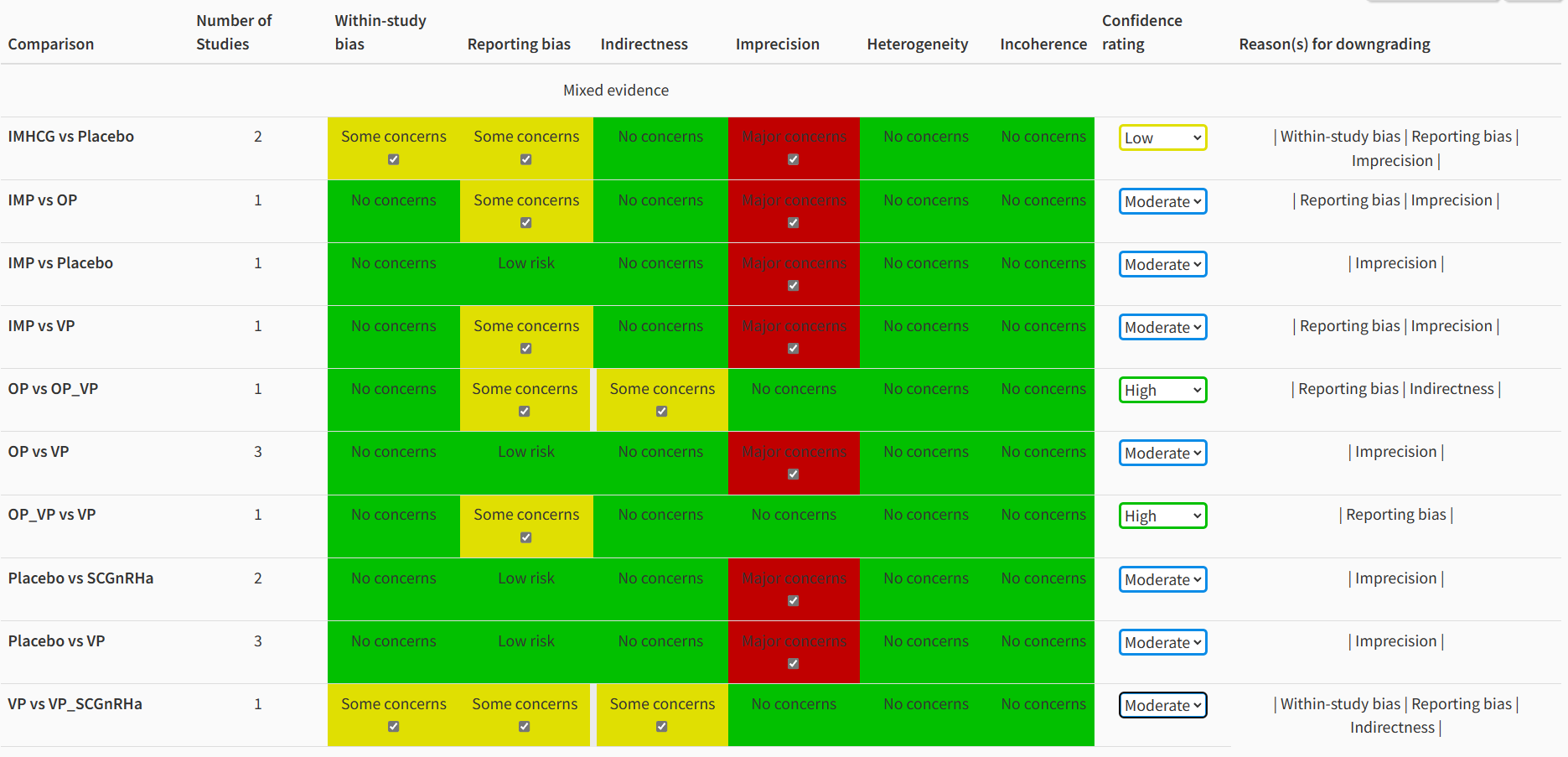


Table S2. CiNeMA Ratings; Mixed Evidence. Clinical Pregnancy.


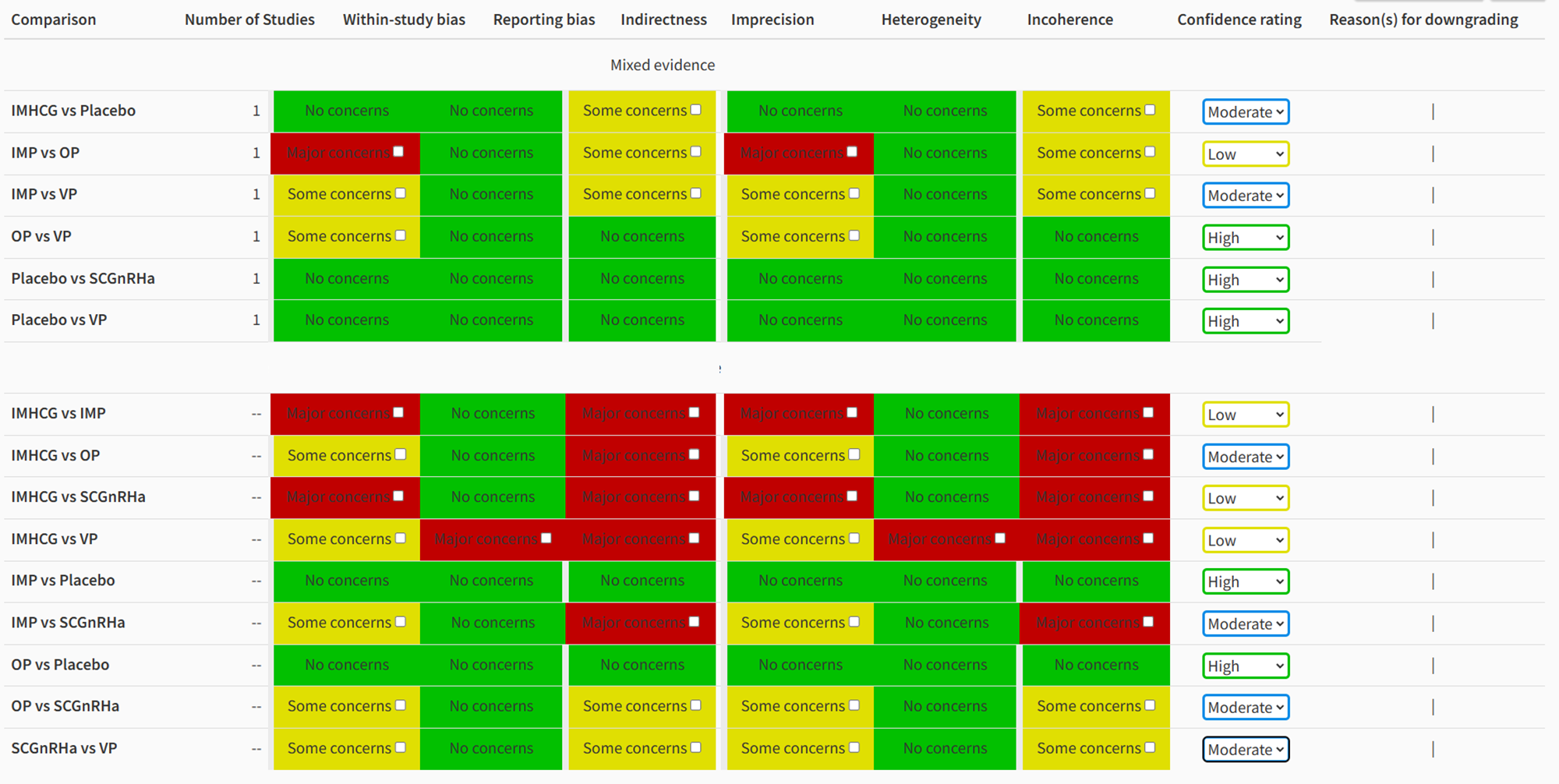


Table S3. CiNeMA Ratings; Mixed Evidence. Live Birth.


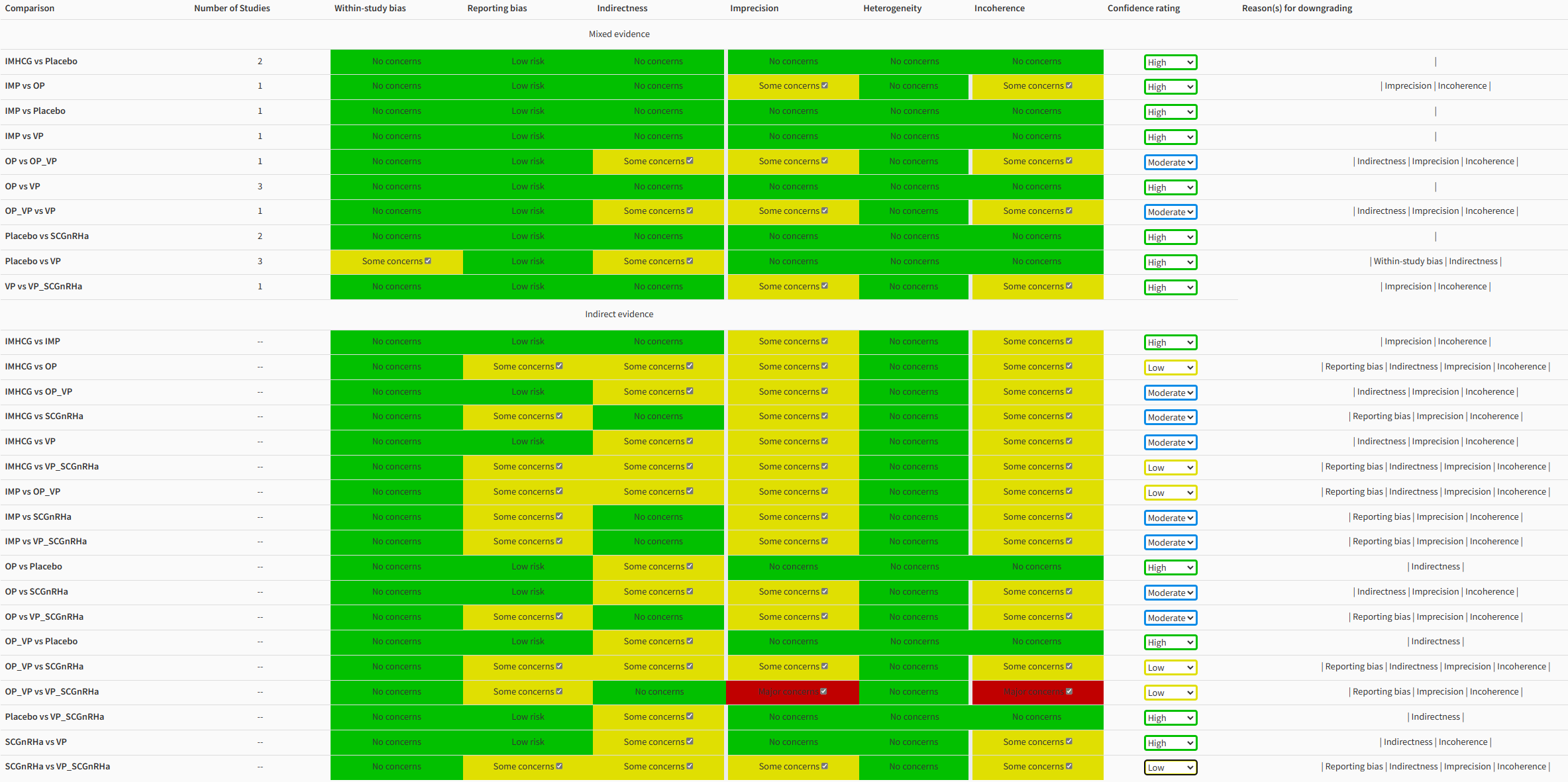


Table S4. CiNeMA Ratings; Mixed Evidence. Miscarriage.


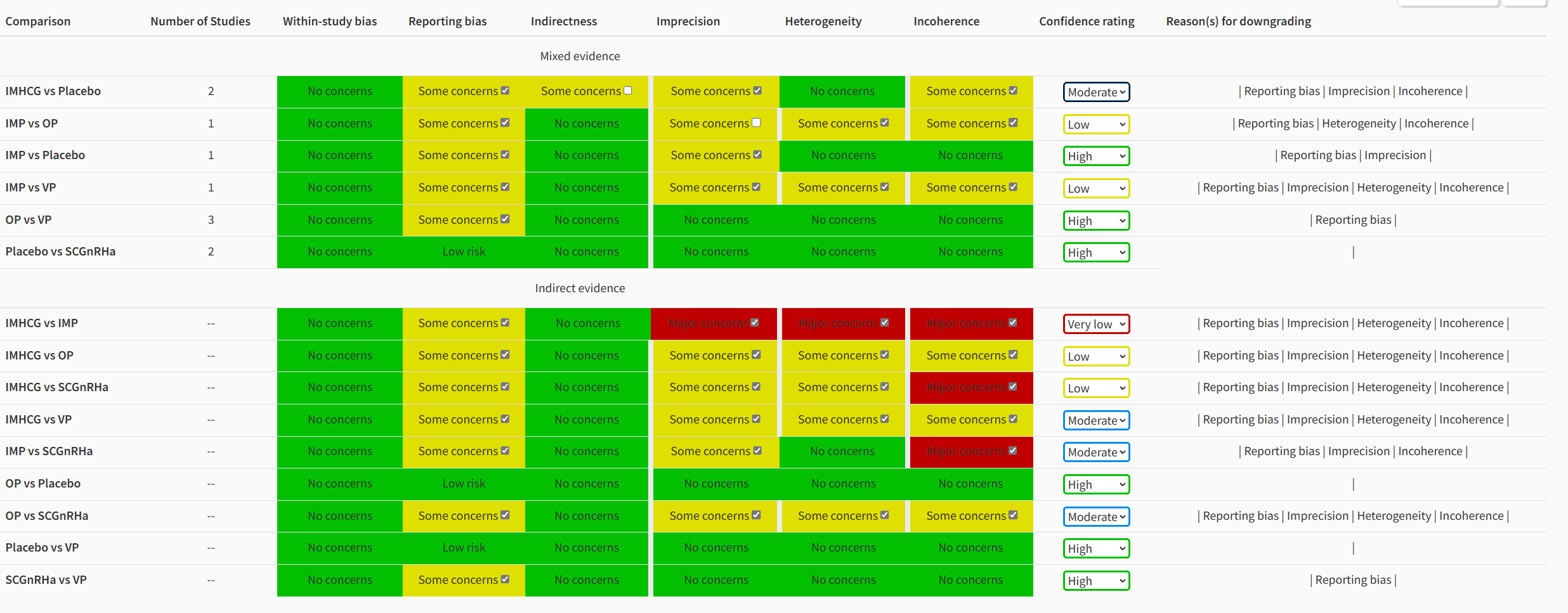


Table S5. CiNeMA Ratings; Mixed Evidence. Biochemical Pregnancy.


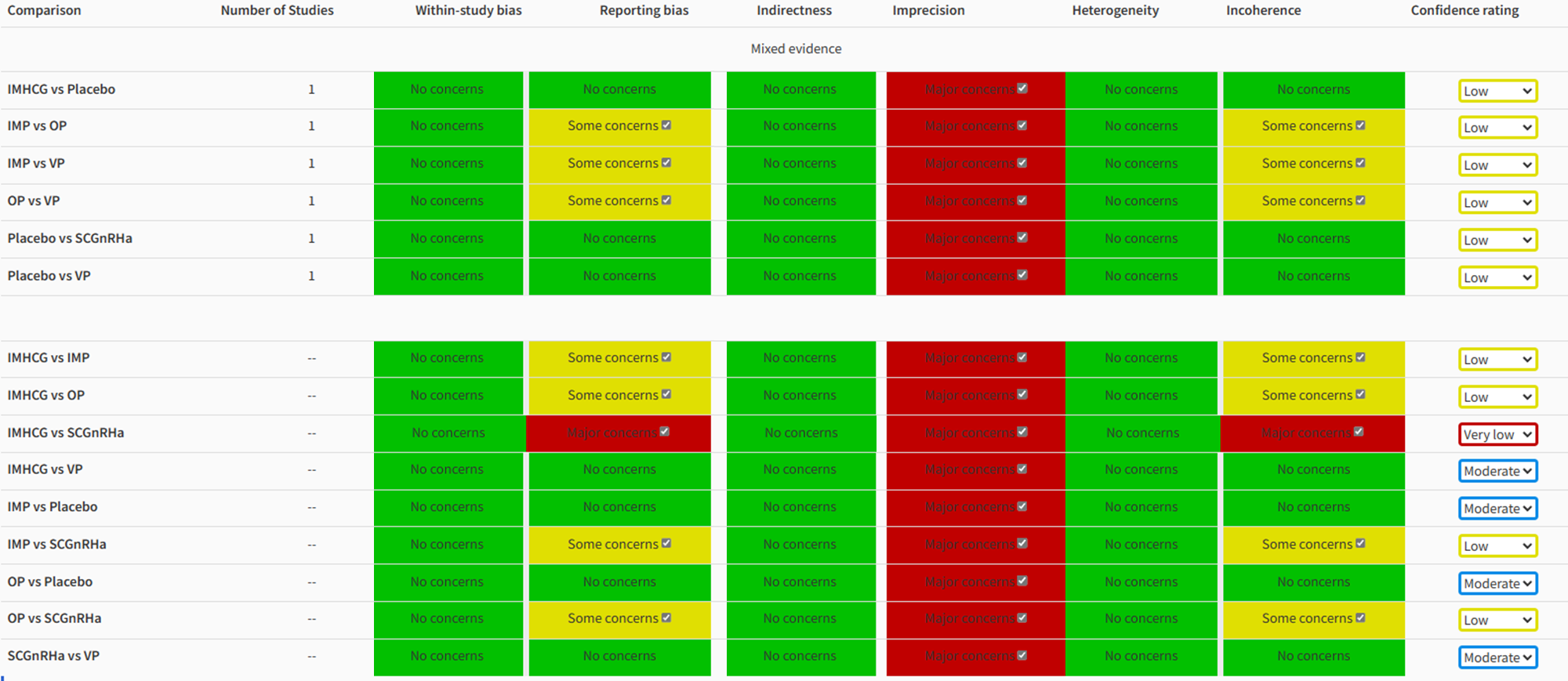


Table S6. CiNeMA Ratings; Mixed Evidence. Multiple Pregnancy.

| **Clinical Pregnancy** | **IMHCG** | **IMP** | **OP** | **OP_VP** | **Placebo** | **SCGnRHa** | **VP** | **VP_SCGnRHa** |
| --- | --- | --- | --- | --- | --- | --- | --- | --- |
| IMHCG | IMHCG | 1.96 (0.74, 5.61) | 1.74 (0.67, 4.12) | 0.65 (0.23, 1.63) | 1.17 (0.72, 1.91) | 1.18 (0.61, 2.48) | 1.53 (0.76, 2.92) | 3.12 (1.11, 8.56) |
| IMP | 0.51 (0.18, 1.35) | IMP | 0.88 (0.27, 2.42) | 0.33 (0.09, 0.96) | 0.59 (0.24, 1.39) | 0.6 (0.22, 1.61) | 0.77 (0.28, 1.91) | 1.57 (0.45, 5.06) |
| OP | 0.57 (0.24, 1.5) | 1.13 (0.41, 3.65) | OP | 0.37 (0.18, 0.76) | 0.67 (0.33, 1.53) | 0.68 (0.29, 1.86) | 0.87 (0.48, 1.67) | 1.76 (0.7, 5.04) |
| OP_VP | 1.54 (0.61, 4.3) | 3.04 (1.04, 10.67) | 2.7 (1.32, 5.45) | OP_VP | 1.8 (0.82, 4.44) | 1.82 (0.74, 5.34) | 2.34 (1.22, 4.89) | 4.81 (1.78, 14.12) |
| Placebo | 0.86 (0.52, 1.38) | 1.68 (0.72, 4.25) | 1.5 (0.65, 3.07) | 0.56 (0.23, 1.22) | Placebo | 1.01 (0.63, 1.74) | 1.3 (0.79, 2.04) | 2.68 (1.05, 6.37) |
| SCGnRHa | 0.85 (0.4, 1.63) | 1.67 (0.62, 4.62) | 1.48 (0.54, 3.42) | 0.55 (0.19, 1.35) | 0.99 (0.57, 1.58) | SCGnRHa | 1.29 (0.6, 2.43) | 2.65 (0.88, 6.97) |
| VP | 0.66 (0.34, 1.32) | 1.29 (0.52, 3.54) | 1.15 (0.6, 2.09) | 0.43 (0.2, 0.82) | 0.77 (0.49, 1.26) | 0.78 (0.41, 1.67) | VP | 2.04 (0.97, 4.41) |
| VP_SCGnRHa | 0.32 (0.12, 0.9) | 0.64 (0.2, 2.25) | 0.57 (0.2, 1.43) | 0.21 (0.07, 0.56) | 0.37 (0.16, 0.95) | 0.38 (0.14, 1.13) | 0.49 (0.23, 1.04) | VP_SCGnRHa |
|  |  |  |  |  |  |  |  |  |
| **Live Birth** | **IMP** | **OP** | **Placebo** | **SCGnRHa** | **VP** |  |  |  |
| IMHCG | IMHCG | 2.81 (0.52, 14.48) | 2.47 (0.47, 12.93) | 1.2 (0.51, 2.79) | 2.05 (0.54, 7.95) | 2.29 (0.63, 8.09) |  |  |
| IMP | 0.36 (0.07, 1.93) | IMP | 0.89 (0.3, 2.6) | 0.43 (0.1, 1.82) | 0.73 (0.12, 4.31) | 0.82 (0.27, 2.39) |  |  |
| OP | 0.4 (0.08, 2.13) | 1.13 (0.38, 3.35) | OP | 0.49 (0.12, 2) | 0.83 (0.14, 4.83) | 0.92 (0.32, 2.66) |  |  |
| Placebo | 0.83 (0.36, 1.96) | 2.33 (0.55, 9.72) | 2.06 (0.5, 8.57) | Placebo | 1.7 (0.6, 4.97) | 1.9 (0.74, 4.91) |  |  |
| SCGnRHa | 0.49 (0.13, 1.85) | 1.37 (0.23, 8.01) | 1.21 (0.21, 7.08) | 0.59 (0.2, 1.67) | SCGnRHa | 1.11 (0.27, 4.55) |  |  |
| VP | 0.44 (0.12, 1.59) | 1.23 (0.42, 3.67) | 1.09 (0.38, 3.12) | 0.53 (0.2, 1.36) | 0.9 (0.22, 3.71) | VP |  |  |
|  |  |  |  |  |  |  |  |  |
| **Miscarriage** | **IMP** | **OP** | **OP_VP** | **Placebo** | **SCGnRHa** | **VP** | **VP_SCGnRHa** |  |
| IMHCG | IMHCG | 0.42 (0.07, 2.37) | 0.95 (0.21, 4.33) | 0.53 (0.1, 3.09) | 0.88 (0.4, 1.97) | 0.83 (0.26, 2.43) | 0.62 (0.19, 2) | 0.4 (0.08, 2.04) |
| IMP | 2.38 (0.42, 14.8) | IMP | 2.25 (0.45, 13.49) | 1.27 (0.21, 9.5) | 2.08 (0.45, 11.18) | 1.96 (0.35, 11.98) | 1.45 (0.32, 7.48) | 0.95 (0.14, 7.09) |
| OP | 1.06 (0.23, 4.8) | 0.44 (0.07, 2.23) | OP | 0.56 (0.18, 2.01) | 0.93 (0.25, 3.42) | 0.87 (0.19, 3.78) | 0.65 (0.24, 1.85) | 0.42 (0.09, 1.91) |
| OP_VP | 1.88 (0.32, 9.73) | 0.79 (0.11, 4.77) | 1.79 (0.5, 5.68) | OP_VP | 1.66 (0.34, 7.04) | 1.53 (0.26, 7.76) | 1.16 (0.3, 3.9) | 0.75 (0.13, 4.06) |
| Placebo | 1.13 (0.51, 2.53) | 0.48 (0.09, 2.22) | 1.08 (0.29, 3.95) | 0.6 (0.14, 2.92) | Placebo | 0.93 (0.42, 1.97) | 0.7 (0.3, 1.68) | 0.45 (0.11, 1.89) |
| SCGnRHa | 1.21 (0.41, 3.8) | 0.51 (0.08, 2.89) | 1.15 (0.26, 5.27) | 0.65 (0.13, 3.86) | 1.07 (0.51, 2.38) | SCGnRHa | 0.75 (0.24, 2.47) | 0.48 (0.1, 2.52) |
| VP | 1.61 (0.5, 5.16) | 0.69 (0.13, 3.11) | 1.54 (0.54, 4.24) | 0.86 (0.26, 3.32) | 1.43 (0.6, 3.37) | 1.34 (0.4, 4.17) | VP | 0.65 (0.2, 2.03) |
| VP_SCGnRHa | 2.51 (0.49, 12.83) | 1.05 (0.14, 7.23) | 2.37 (0.52, 11.18) | 1.34 (0.25, 7.81) | 2.21 (0.53, 9.13) | 2.08 (0.4, 10.2) | 1.55 (0.49, 4.92) | VP_SCGnRHa |
|  |  |  |  |  |  |  |  |  |
| **Biochemical Pregnancy** | **IMP** | **OP** | **Placebo** | **SCGnRHa** | **VP** |  |  |  |
| IMHCG | IMHCG | 1.43 (0.55, 3.78) | 1.34 (0.2, 13.51) | 1.19 (0.82, 1.73) | 1.25 (0.73, 2.15) | 0.92 (0.13, 9.84) |  |  |
| IMP | 0.7 (0.26, 1.81) | IMP | 0.93 (0.17, 8.07) | 0.84 (0.33, 1.99) | 0.88 (0.32, 2.25) | 0.65 (0.11, 5.93) |  |  |
| OP | 0.75 (0.07, 5.07) | 1.08 (0.12, 5.78) | OP | 0.9 (0.09, 5.82) | 0.93 (0.09, 6.23) | 0.7 (0.37, 1.29) |  |  |
| Placebo | 0.84 (0.58, 1.22) | 1.2 (0.5, 3.03) | 1.12 (0.17, 11) | Placebo | 1.05 (0.72, 1.55) | 0.78 (0.11, 8.14) |  |  |
| SCGnRHa | 0.8 (0.46, 1.37) | 1.14 (0.44, 3.1) | 1.07 (0.16, 11.13) | 0.95 (0.64, 1.39) | SCGnRHa | 0.74 (0.1, 8.26) |  |  |
| VP | 1.09 (0.1, 7.73) | 1.54 (0.17, 9.04) | 1.43 (0.77, 2.69) | 1.28 (0.12, 9.14) | 1.34 (0.12, 9.77) | VP |  |  |
|  |  |  |  |  |  |  |  |  |
| **Multiple Pregnancy** | **IMP** | **OP** | **Placebo** | **SCGnRHa** | **VP** |  |  |  |
| IMHCG | IMHCG | 3.61 (0.05, 284.57) | 6.77 (0.18, 481.05) | 0.92 (0.17, 4.86) | 1.27 (0.18, 8.59) | 2.57 (0.22, 38.6) |  |  |
| IMP | 0.28 (0, 22.04) | IMP | 1.88 (0.12, 64.14) | 0.26 (0, 15.88) | 0.35 (0.01, 23.24) | 0.74 (0.02, 30.4) |  |  |
| OP | 0.15 (0, 5.55) | 0.53 (0.02, 8.12) | OP | 0.14 (0, 3.43) | 0.19 (0, 5.4) | 0.4 (0.01, 5.73) |  |  |
| Placebo | 1.09 (0.21, 5.81) | 3.88 (0.06, 239.52) | 7.26 (0.29, 396.12) | Placebo | 1.38 (0.52, 3.64) | 2.72 (0.49, 24.48) |  |  |
| SCGnRHa | 0.79 (0.12, 5.46) | 2.82 (0.04, 192.93) | 5.36 (0.19, 315.83) | 0.72 (0.27, 1.92) | SCGnRHa | 1.98 (0.27, 21.36) |  |  |
| VP | 0.39 (0.03, 4.54) | 1.34 (0.03, 53.66) | 2.51 (0.17, 89.36) | 0.37 (0.04, 2.05) | 0.5 (0.05, 3.68) | VP |  |  |

Table S7. Relative treatment effects in ranked order for all studies, consistency assessment of direct and indirect OR for primary outcomes (Clinical Pregnancy, Live Birth, Miscarriage) and secondary (Biochemical Pregnancy and Multiple pregnancy) outcomes.
