# Supplementary material for: Comparison of Luteal Support Protocols in Frozen IVF/ICSI Cycles: A Network Meta‐Analysis
Source: BJOG. 2025 May 2;132(9):1187–201. doi: 10.1111/1471-0528.18172 (PMC12232591; doi:10.1111/1471-0528.18172)
Supplement: Supplementary file 2 — Figures S1–S10. [file BJO-132-1187-s002.docx]

**Fig. S1.** Population percentage and crude numbers exposed to each luteal support regimen and baseline embryological and IVF/ICSI specific characteristics. Comparison of median percentage of day 5 embryo transfers [%] (A), grade of embryos transferred (% of good quality embryos) (B), distribution of cycle type category across included participants [N,%] (C), Follow up duration per LPS protocol (months) [95% CI] (D).

**Figure S2.** Population percentage and crude numbers exposed to each luteal support regimen and baseline biochemical characteristics. Comparison of median estradiol on the day of HCG trigger [pg/ml] (A), median progesterone on the day of HCG trigger [ng/ml] (B), distribution of IVF vs. ICSI assisted conception [N, %] (C), Ovarian stimulation protocol GnRH antagonist vs. agonist [N, %] (D).

**Fig. S3.** Percentage rate and crude numbers of fertilisation rate [%] (A) and number of oocytes retrieved [Median; 95%CI] (B) per luteal support regimen cohort.


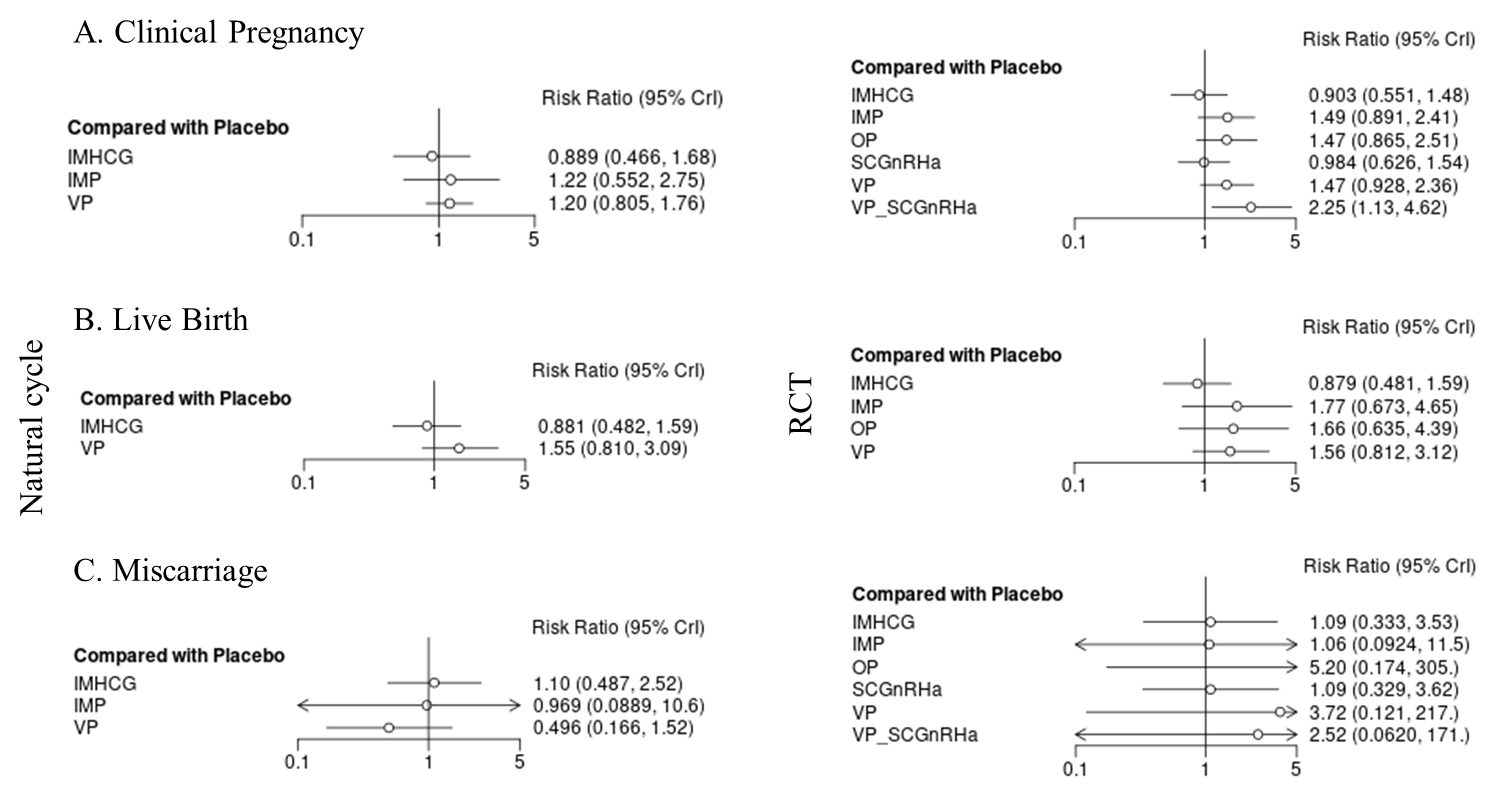


**Fig. S4.** Luteal support Bayesian random effect consistency forest plot (Risk ratio, 95% CrI) for (A) Clinical Pregnancy (B) Live Birth (C) Miscarriage outcomes. Subgroup analysis on natural cycles only and RCTs.

*
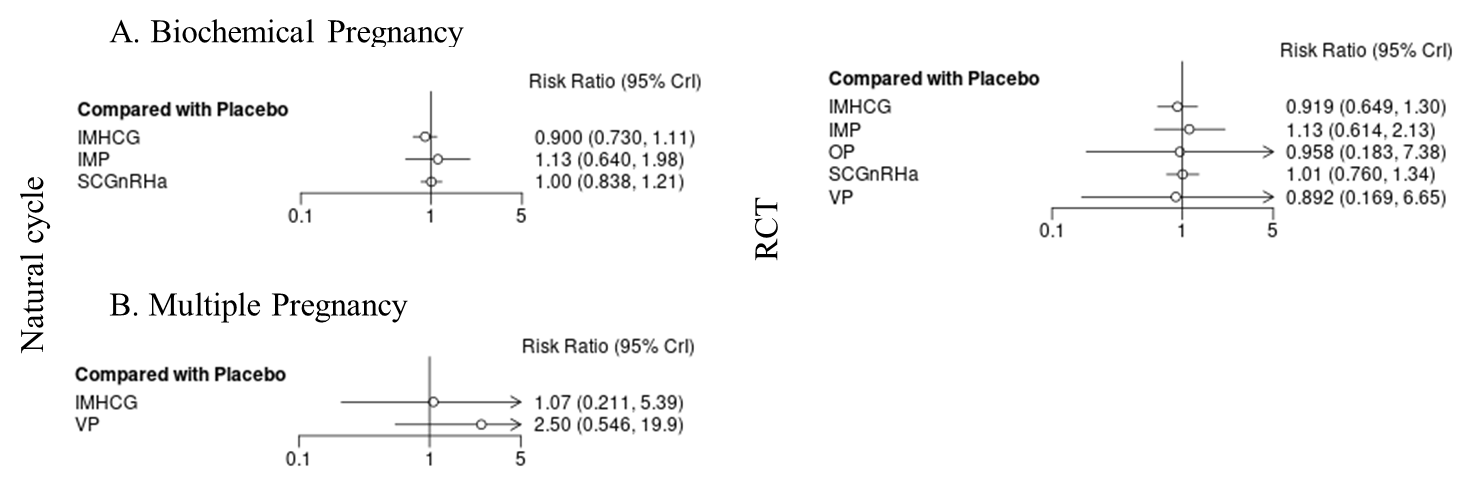
*

**Fig. S5.** Luteal support Bayesian random effect consistency forest plot (Risk ratio, 95% CrI) for Biochemical Pregnancy (A) and Multiple pregnancy (B) outcomes. Subgroup analysis on natural cycles only and RCTs.

*
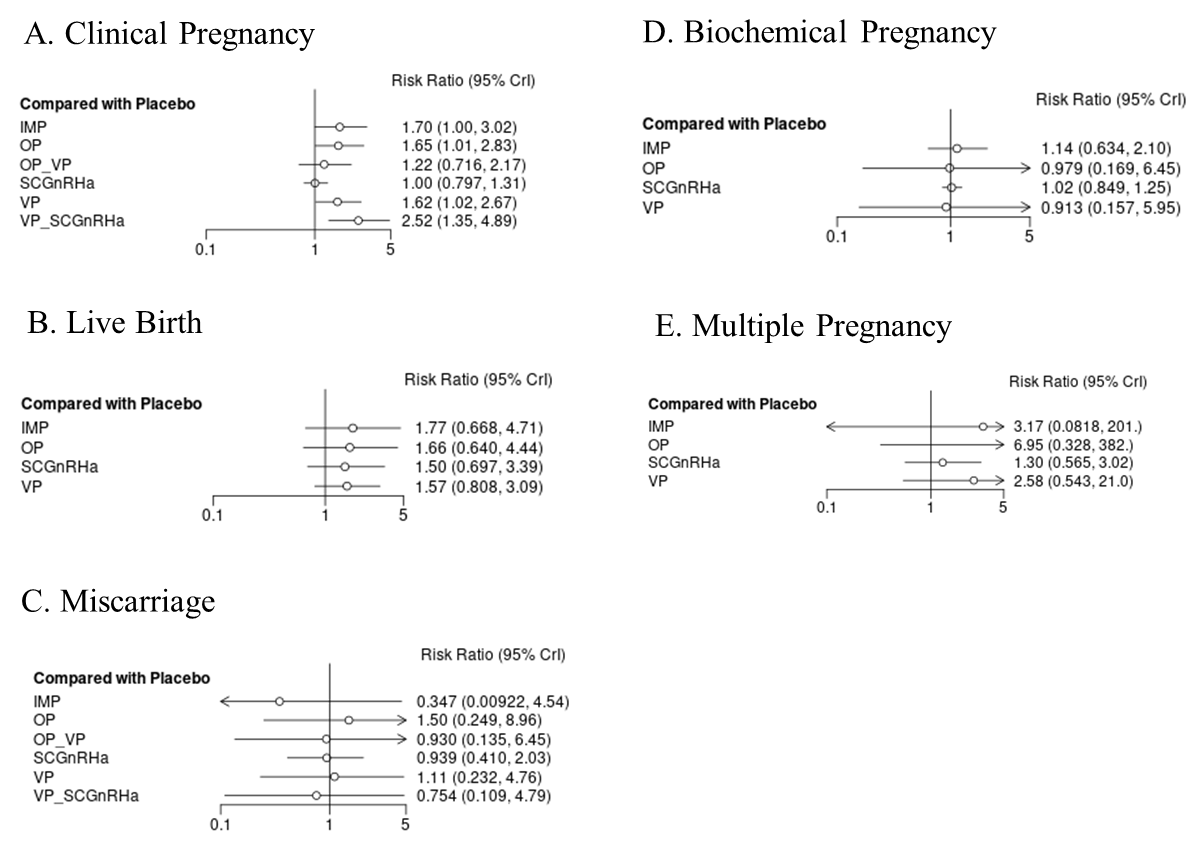
*

**Fig. S6.** Luteal support Bayesian random effect consistency forest plot (Risk ratio, 95% CrI) for (A) Clinical Pregnancy (B) Live Birth (C) Miscarriage outcomes (D) Biochemical pregnancy (E.) Multiple Pregnancy. Subgroup analysis on medicated cycles only.


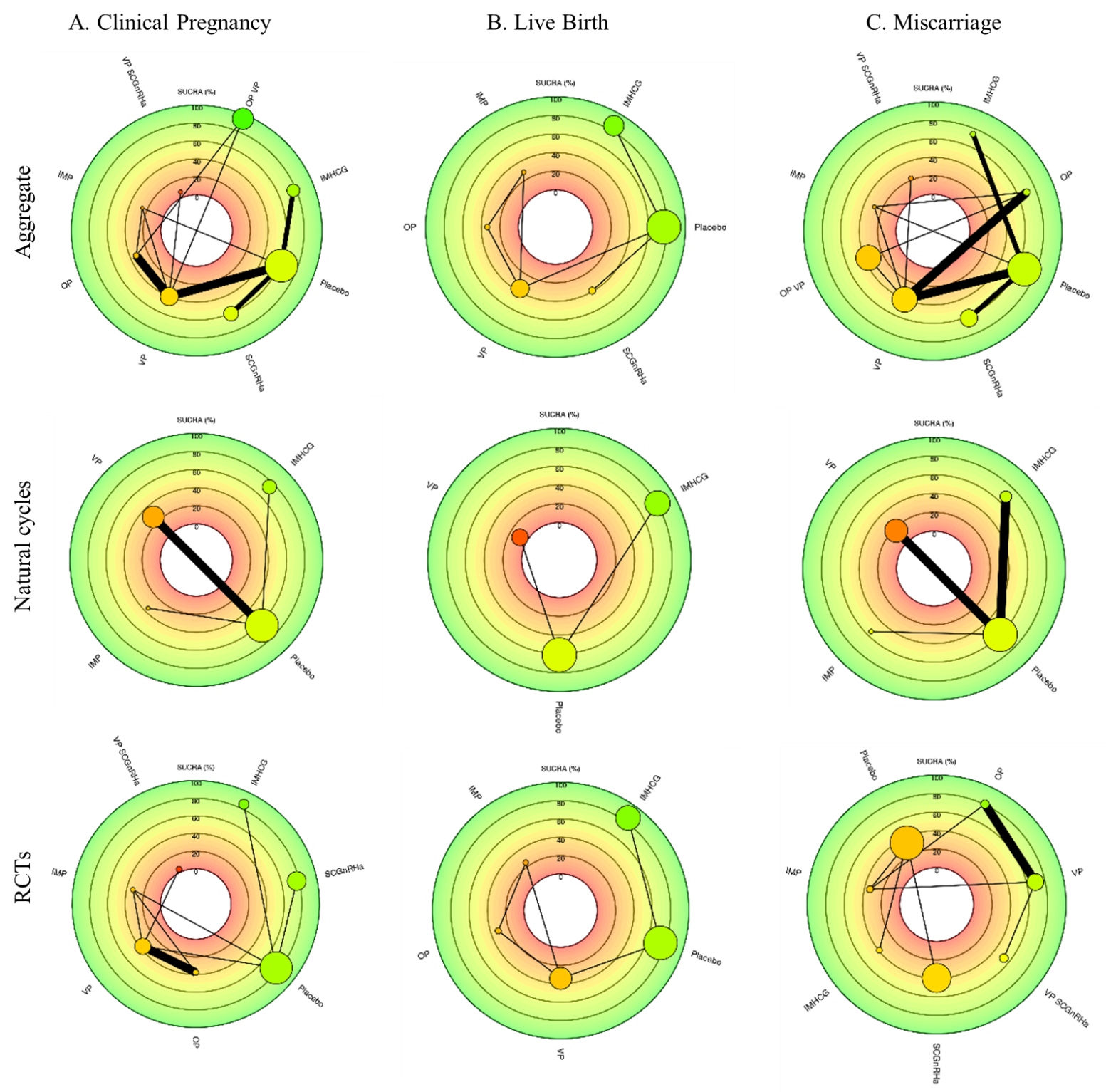


**Fig.S.7.** Radial SUCRA plot of NMA included LPS protocols. Clinical pregnancy (A), Live Birth (B), Miscarriage (C) for aggregate and subgroup analysis.


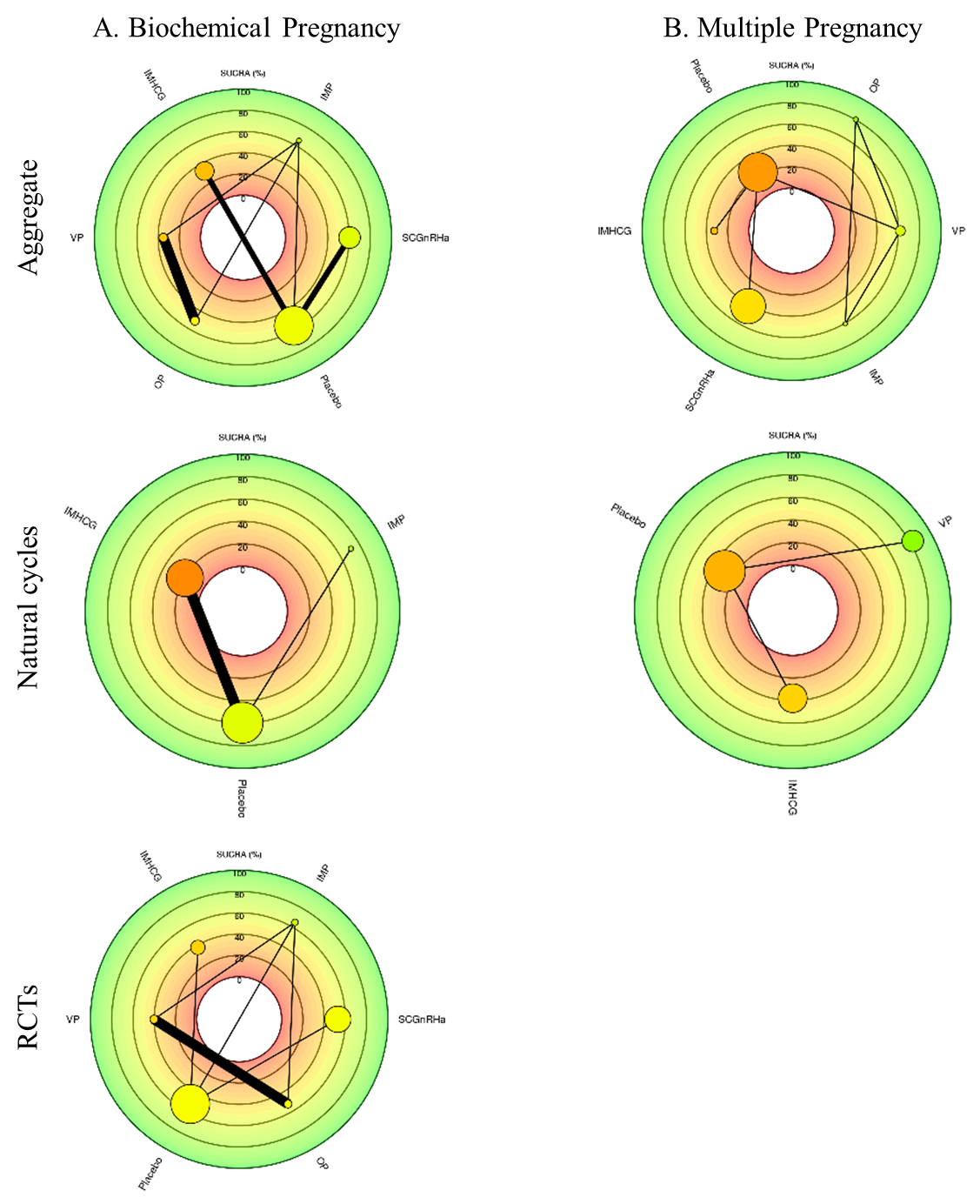


**Fig.S.8.** Radial SUCRA plot of NMA included LPS protocols. Biochemical pregnancy (A), Multiple Pregnancy (B) for aggregate and subgroup analysis.


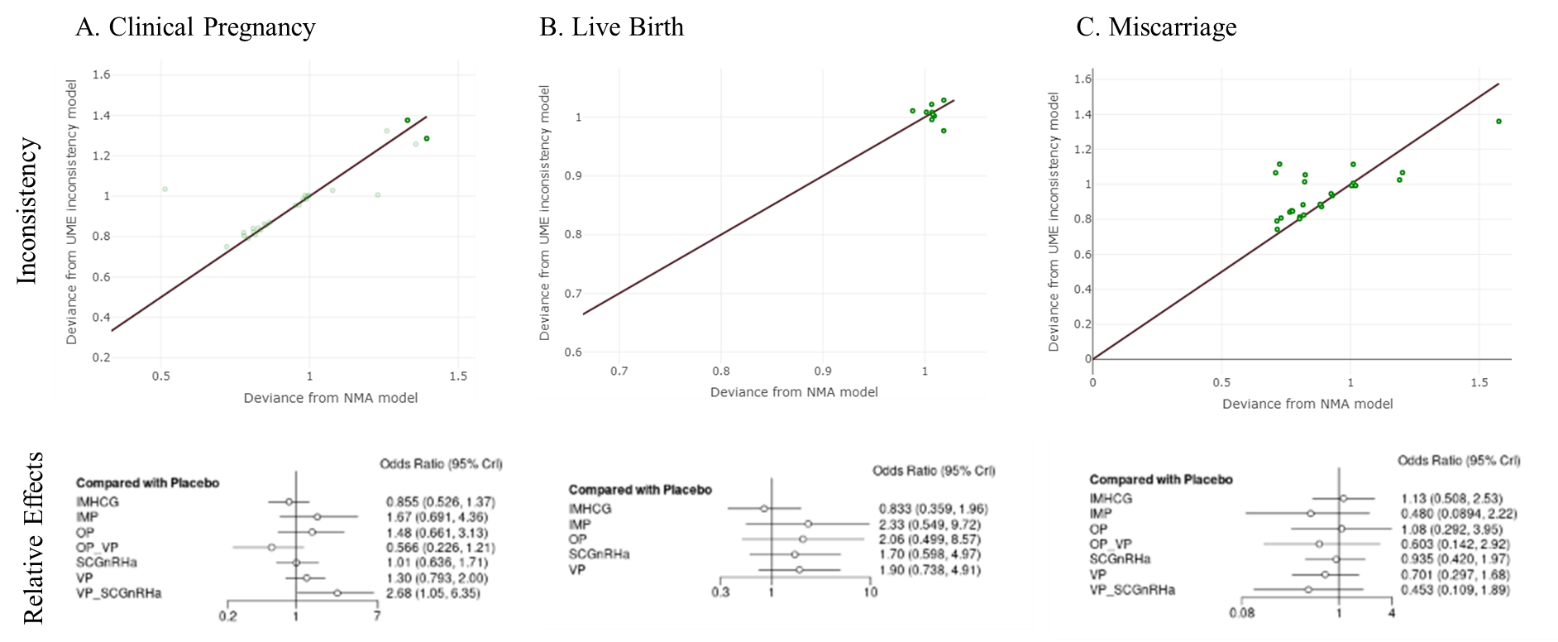


**Fig. S9** Residual deviance from NMA model and UME inconsistency model for all studies. Clinical pregnancy (A), Live Birth (B), Miscarriage (C) and Relative effects [OR, 95%CI].

**
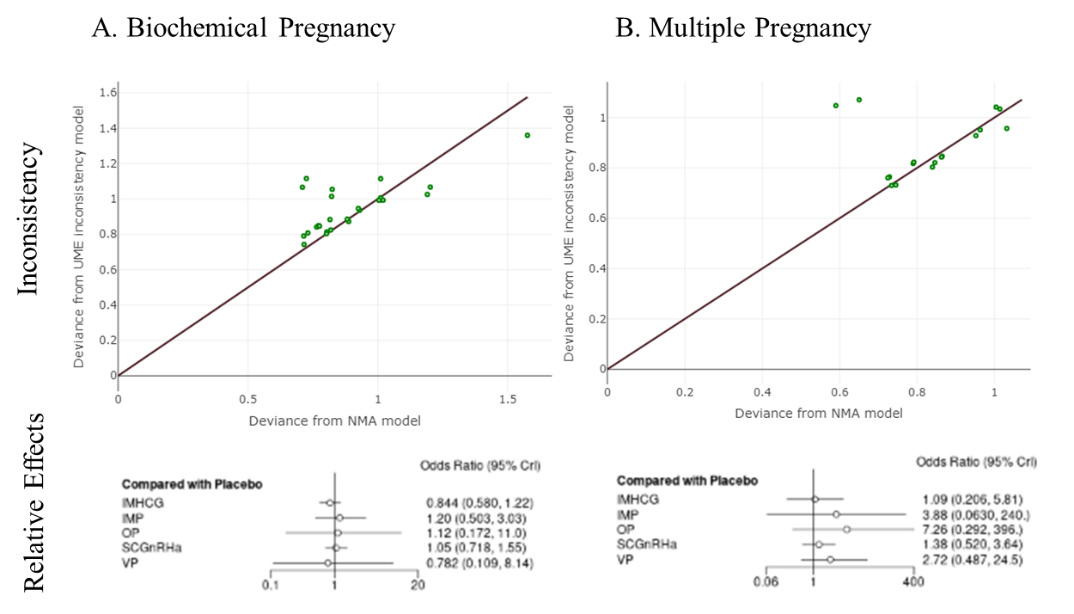
**

**Fig. S10** Residual deviance from NMA model and UME inconsistency model for all studies. Biochemical pregnancy (A), Multiple Pregnancy (B), and Relative effects [OR, 95%CI].
